# Metabolites of Marine Sediment-Derived Fungi: Actual Trends of Biological Activity Studies

**DOI:** 10.3390/md19020088

**Published:** 2021-02-04

**Authors:** Anton N. Yurchenko, Elena V. Girich, Ekaterina A. Yurchenko

**Affiliations:** 1 Laboratory of Chemistry of Microbial Metabolites, G.B. Elyakov Pacific Institute of Bioorganic Chemistry, Far Eastern Branch of Russia Academy of Sciences, 690022 Vladivostok, Russia; yurchenkoan@piboc.dvo.ru (A.N.Y.); ev.girich@piboc.dvo.ru (E.V.G.); 2 Laboratory of Bioassays and Mechanisms of Action of Biologically Active Substances, G.B. Elyakov Pacific Institute of Bioorganic Chemistry, Far Eastern Branch of Russia Academy of Sciences, 690022 Vladivostok, Russia

**Keywords:** marine sediments, fungi, secondary metabolites, antiviral activity, antimicrobial activity, cytotoxic activity, cytoprotective activity

## Abstract

Marine sediments are characterized by intense degradation of sedimenting organic matter in the water column and near surface sediments, combined with characteristically low temperatures and elevated pressures. Fungi are less represented in the microbial communities of sediments than bacteria and archaea and their relationships are competitive. This results in wide variety of secondary metabolites produced by marine sediment-derived fungi both for environmental adaptation and for interspecies interactions. Earlier marine fungal metabolites were investigated mainly for their antibacterial and antifungal activities, but now also as anticancer and cytoprotective drug candidates. This review aims to describe low-molecular-weight secondary metabolites of marine sediment-derived fungi in the context of their biological activity and covers research articles published between January 2016 and November 2020.

## 1. Introduction

Marine sediments are extreme marine environmental conditions reflecting the condition of sea waters as well as coastal areas. A wide variety of inhabitants of marine sediments makes this ecosystem highly competitive, which affects the metabolism of organisms.

Marine sediments are characterized by intense degradation of sedimenting organic matter in the water column and near surface sediments, resulting in recalcitrant organic matter in subsurface layers, combined with characteristically low temperatures and elevated pressures. For a long time, these geophysical conditions were thought to make this area uninhabitable. The perceived low energy supply coupled with geological time scales resulted in the view that most microorganisms in sub seafloor sediments must be either inactive or adapted for extraordinarily low metabolic activity [1]. The revision of the microbiological methods for studying sediments has led to the modern view that the biomass of marine sediments, including deep-sea sediments, can be very significant and have a serious impact on the geological processes of the ocean bottom [2]. The microbial community of bottom sediments includes bacteria and archaea as well as fungi, but the role of the latter in the marine carbon cycle and other biogeochemical process is not yet clear enough [2].

The few studies devoted to a comprehensive estimation of benthic biodiversity, including both prokaryotic and eukaryotic microorganisms, show that fungi (eukaryotic microorganisms) are less represented in the microbial communities of sediments than bacteria and archaea (prokaryotes). A study of microbial communities of coral reefs in the Gulf of Thailand (the South China Sea) showed that relative abundance of fungi in bottom sediments was only 0.017% (avg.), whereas in coral samples it was 25–96 times more [3]. 

The diversity of microorganisms, both prokaryotic and eukaryotic, of Stellwagen Bank National Marine Sanctuary in the Gulf of Maine (the Atlantic Ocean, US coastline) has been investigated. The majority of the community was bacterial, with 59 phyla, but also represented were 17 Fungi, 9 Archaea, 18 Animalia, 14 Chromista, 8 Protozoa, and 2 Plantae phyla [4].

Microeukaryotes (fungi) and prokaryotes (bacteria and archaea) are in a permanent interaction inside sediment ecosystems and inevitably influence each other. 

Bacteria produce antifungal metabolites to regulate the number of fungi in communities [5]. In turn, fungi release antibacterial compounds in an environment.

The fungus–bacterium symbiont SCSIO F190/B001 was isolated from a marine sediment sample. The 16S rRNA gene sequence showed a high degree of similarity (99%) to the bacterium *Alcaligenes faecalis* whereas the ITS1-5.8S rDNA-ITS2 sequence showed 96% sequence similarity and 93% query coverage to fungal *Spiromastix* sp. CBS13827 sequences identifiable by BLASTN. The bacterial symbiont modulates the fungal host to biosynthesize an antimicrobial polyketide spiromarmycin. Spiromarmycin appears to endow upon the symbiont pair a protective/defensive means of warding off competitor organisms, be they prokaryotic or eukaryotic microorganisms [6].

The biosynthetic pathways in marine fungi including marine sediment-derived fungi result in mycotoxins targeting both bacterial and eukaryotic components of bottom sediment communities [7,8,9]. In the past 15 years, marine sediment-derived fungi have been one of the leading sources of new secondary metabolites that exhibit various types of biological activity. However, for a long time, the study of their biological activity has focused almost exclusively on mycotoxins.

In total, 346 compounds have been described from marine sediment-derived fungi from 2005 till 2015. Antiviral and antimicrobial activity (including sortase A and inhibition of some other enzymes) has been reported for 6 and 24 compounds, respectively. Cytotoxic activity together with cancer-preventive activity and indolamino-2,3-dioxygenase activity inhibition was described for 127 metabolites. At the same time, only nine compounds were reported as anti-inflammatory agents, as well as acetylcholinesterase inhibitors and neuritogenic inductors [10,11,12,13]. 

Few reviews focus on metabolites of marine fungi in general or producers from particular taxa. However, to the best of our knowledge there are no reviews focused on the metabolites of marine sediment-derived fungi.

This review focuses on low-molecular-weight secondary metabolites of marine sediment-derived fungi in the context of their biological activity, and covers research articles published between January 2016 and November 2020.

## 2. Antiviral Activity

The COVID-19 pandemic that was declared by World Health Organization (WHO) on March 11, 2020 has drawn a lot of attention to drugs with a direct antiviral effect against various viruses, not just molecules that induce antiviral immunity. Despite the increasing spread of antiviral therapy with monoclonal antibodies, the majority of antiviral drugs are low-molecular-weight compounds belonging to various chemical classes. This maintains a stable interest in the study of antiviral properties of the metabolites of marine fungi [14].

**Figure 1:**
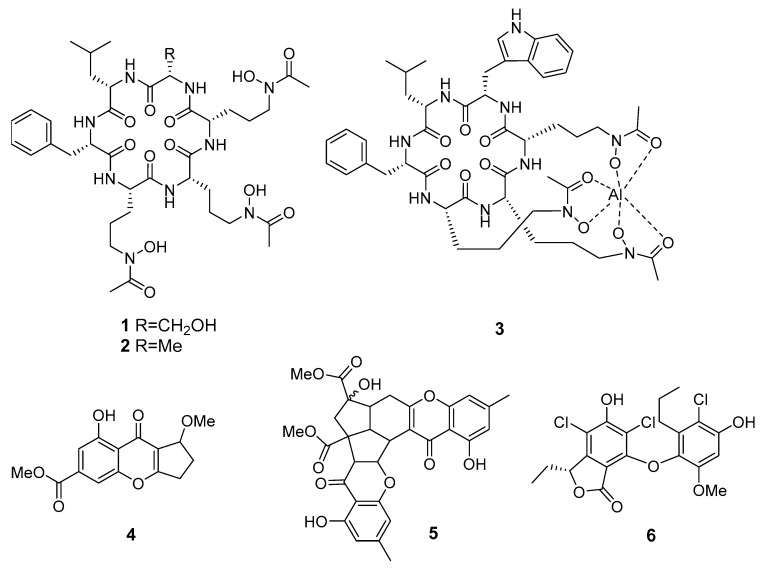
The antiviral metabolites of marine sediment-derived fungi.

The new hydroxamate siderophore-containing cyclopeptides acremonpeptides A–D and known Al(III)−acremonpeptide D (Figure 1) were isolated from the marine fungus *Acremonium persicinum* SCSIO115 (from the South China Sea). Acremonpeptides A (**1**) and B (**2**) and compound **3** have shown moderate antiviral effect against the herpes simplex virus 1 [15].

The xanthone derivatives coniochaetone J (**4**) and epiremisporine B (**5**) from *Penicillium* sp. SCSIO Ind16F01 (the Indian Ocean) exhibited weak activity against enterovirus EV71 in vitro, and **5** also was active against influenza A virus subtype H3N2. Moreover, these metabolites have shown cytotoxic effects on the human erythroleukemia K562, human breast MCF-7, and human gastric carcinoma SGC7901 cancer cell lines [16].

A new class of phenolic lactones spiromastilactones A–M was isolated from a deep-sea derived fungus *Spiromastix* sp. (south of the Atlantic Ocean). Most of these compounds exert inhibitory activity against WSN influenza virus with low cytotoxicity. Moreover, spiromastilactone D (**6**), a 5′-chloro-2′-methoxy substituted analogue, displayed the most potential to inhibit a panel of influenza A and B viruses including oseltamivir- and amantadine-resistant strains [17].

## 3. Antibacterial Activity

Since the isolation of penicillin, the antibacterial activity of metabolites of microfilamentous fungi has been the focus of investigations. Marine fungi are no exception in this sense. From 1998 to 2019, more than 270 compounds with antimicrobial properties were isolated from various marine-derived fungi [18].

**Figure 2:**
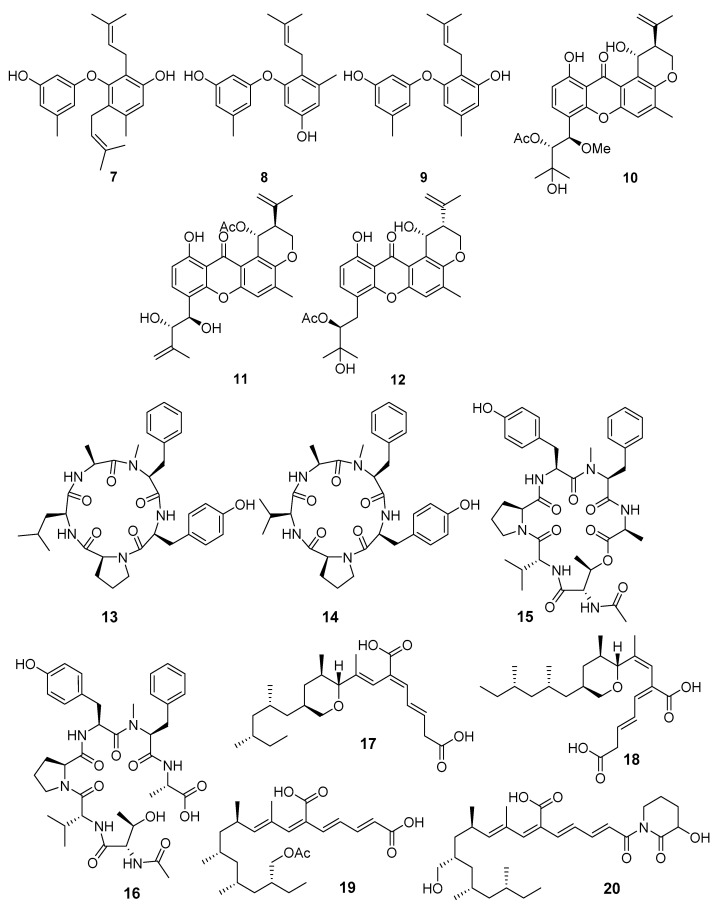
The antibacterial metabolites (**7**–**20**) of marine sediment-derived fungi.

A new diphenyl ether, diorcinol K (Figure 2), along with three known compounds, diorcinols D, F and I were isolated from *Aspergillus* sp. CUGB-F046 strain (the Bohai Sea, China). Diorcinol K (**7**), D (**8**) and I (**9**) displayed significant antibacterial activities against *Staphylococcus aureus* and methicillin-resistant *S. aureus* (MRSA) [19].

New prenylxanthones aspergixanthones A–K were isolated from *Aspergillus* sp. ZA-01 (Bohai Sea). Aspergixanthone H (**11**) exhibited strong activity against *Micrococcus lysodeikticus,* while aspergixanthone I (**12**) showed the strongest activity against *Vibrio parahemolyticus*, *V. anguillarum*, and *V. alginolyticus*. Moreover, aspergixanthone G (**10**) showed cytotoxicity against the A-549 cell line [20,21].

Four new peptides were isolated from fungi *Aspergillus allahabadii* and *A. ochraceopetaliformis* (Jeju Island, Korea). Two compounds (**13** and **14**) from *A. allahabadii* were determined to be cyclopentapeptides, while those from *A. ochraceopetaliformis* were a structurally related cyclodepsihexapeptide (**15**) and its linear analogue (**16**). These new compounds exhibited moderate inhibition against the bacterial enzyme sortase A as well as a weak inhibition against isocitrate lyase [22].

Fiscpropionates A–F, new polypropionate derivatives, were isolated from *Aspergillus fischeri* FS452 (Indian Ocean). Four of them, fiscpropionates **17**–**20**, exhibited significant noncompetitive inhibitory activities against *Mycobacterium tuberculosis* protein tyrosine phosphatase B (MptpB) [23]. 

**Figure 3:**
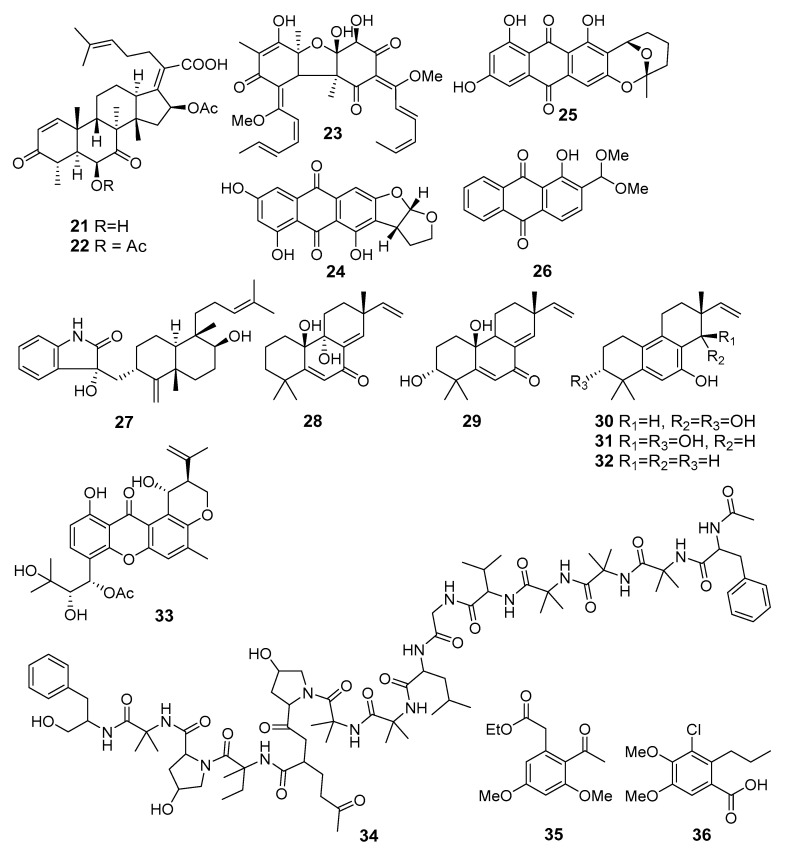
The antibacterial metabolites (**21**–**36**) of marine sediment-derived fungi.

Strong activity against *Staphylococcus aureus* and *E. coli* was reported for helvolinic acid (**21**) and helvolic acid (**22**) (Figure 3) from *Aspergillus fumigatus* MF071 (the Bohai Sea). A genomic data analysis revealed the putative biosynthetic gene clusters *ftm* for fumitremorgins, *pso* for pseurotins, *fga* for fumigaclavines, and *hel* for helvolinic acid. These putative biosynthetic gene clusters fundamentally underpinned the enzymatic and mechanistic function study for the biosynthesis of these compounds [24].

A new sorbicillinoid bisvertinolone (**23**) from *Aspergillus protuberus* MUT3638 (Hammerfest fiord, the Barents Sea) display significant activity against *S. aureus* [25].

New brevianamide diketopiperazines together with known anthraquinones versicolorin B (**24**) and averufin (**25**) were isolated from *Aspergillus versicolor* MF180151 (the Bohai Sea, China). Compounds **24** and **25** showed moderate activities against *S. aureus* and MRSA [26].

A novel anthraquinone, 2-(dimethoxymethyl)-1-hydroxyanthracene-9,10-dione (**26**), was isolated from *Aspergillus versicolor* (West Pacific Ocean) and exhibited strong inhibitory activities against two strains of MRSA. Moreover, this one can inhibit topoisomerase IV and AmpC β-lactamase enzyme activities, shown through molecular docking studies [27].

A new indoloditerpene (**27**) and fifteen known compounds were isolated from *Aspergillus versicolor* ZZ761 (Shengsi Island, the East China Sea). Compound **27** showed antimicrobial activities against *Escherichia coli* and *Candida albicans* [28]. 

Five new 20-nor-isopimarane diterpenoids, aspewentins D–H (**28**–**32**), were isolated from *Aspergillus wentii* SD-310 (the South China Sea). Compounds **28**–**32** showed inhibitory activity against the aquatic pathogens *Edwardsiella tarda*, *Micrococcus luteus*, and human pathogens *Pseudomonas aeruginosa*, *Vibrio harveyi*, and *V. parahemolyticus*. Moreover, compounds **28** and **31** showed activity against the plant pathogen *Fusarium graminearum* [29].

A new emerixanthone E (**33**), together with four known emodin derivatives from *Emericella* sp. (the South China Sea), were isolated and showed moderate antibacterial activities against *E. coli*, *Klebsiella pneumoniae*, *Staphylococcus aureus*, *Enterococcus faecalis*, *Acinetobacter baumannii*, and *Aeromonas hydrophila* [30].

Emerimicin IV (**34**), a unique fungal peptaibol, was isolated from *Emericellopsis minima* (Talcahuano Bay, Chile) and showed bacteriostatic activity against clinical isolates of MRSA and vancomycin-resistant *Enterococcus faecalis* (VRE) [31].

Two new benzoate derivatives, ethyl 3,5-dimethoxy-2-propionylbenzoate (**35**) and ethyl 3,5-dihydroxy-2-propionylbenzoate, and one new phenylacetate derivative, ethyl 3,5-dimethoxy-2-propionylphenylacetate, were isolated from *Engyodontium album* (the Pacific Ocean). Compound **35** exhibited inhibitory activities against MRSA and *Vibrio vulnificus* [32]. Moreover, engyodontiumin A (**36**), a new benzoic acid derivative, was isolated from this fungal strain and showed moderate antibacterial activity against MRSA, *Vibrio vulnificus*, *V. rotiferianus*, and *V. campbellii*, as well as antifungal activity against *Aspergillius niger* [33].

**Figure 4:**
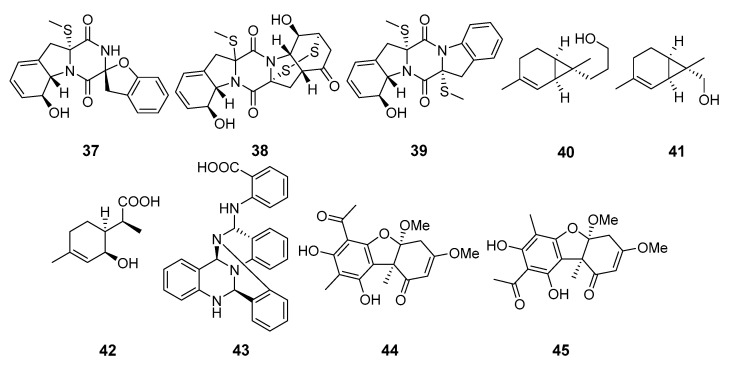
The antibacterial metabolites (**37**–**45**) of marine sediment-derived fungi.

Six new thiodiketopiperazine alkaloids, eutypellazines N-S, were isolated from fungus *Eutypella* sp. MCCC 3A00281 (the South Atlantic Ocean). The eutypellazines P–R (**37**–**39**) (Figure 4) exhibited moderate inhibitory effects against *Staphylococcus aureus* and VRE [34].

Eutypellol A (**40**), the first norsesquiterpenoid of the sequicarene family, as well as eutypellol B (**41**), a rare 7-methyl oxidized 2-carene derivative, and new 2-(2-hydroxy-4-methylcyclohex-3-enyl)propanoic acid (**42**), along with eight known terpenoids, were isolated from *Eutypella scoparia* FS46 (the South China Sea). Compounds **40** and **41** showed a weak antibacterial activity against *S. aureus* [35].

A new alkaloid, a tris-anhydrotetramer of anthranilic acid (α-aminobenzoic acid), named oxysporizoline (**43**), from *Fusarium oxysporum* (Suncheon Bay, South Korea, Korea Strait), showed a weak activity against MRSA and multidrug-resistant *S. aureus* (MDRSA) [36].

Mycousfurans A (**44**) and B (**45**), two new usnic acid congeners, were isolated from *Mycosphaerella* sp. (Donghae-si, Gangwon-do, South Korea, the Japanese Sea) and showed moderate activity against *Kocuria rhizophila* and *S. aureus* [37].

**Figure 5:**
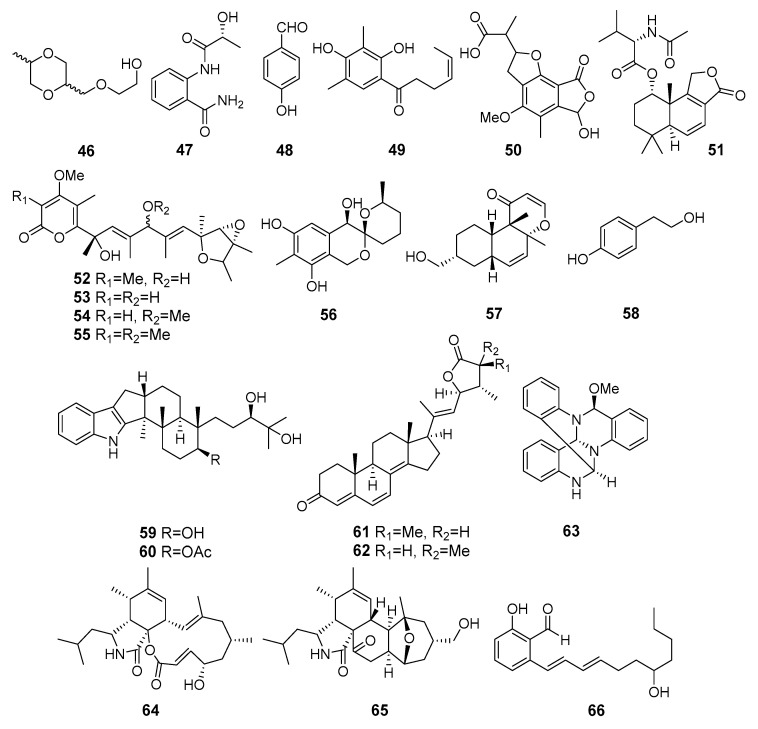
The antibacterial metabolites (**46**–**66**) of marine sediment-derived fungi.

Known 2-[(5-methyl-1,4-dioxan-2-yl)methoxy]ethanol (**46**), 2-[(2R-hydroxypropanoyl)amino]benzamide (**47**), 4-hydroxybenzandehyde (**48**), and 2′,3′-dihydrosorbicillin (**49**) (Figure 5) were isolated from *Penicillium* sp. M30 strain (Con Co Island, the South China Sea). Compound **46** showed a strong inhibitory effect against *Enterococcus faecalis*, whereas both **47** and **48** selectively inhibited *Escherichia coli*. Moreover, 2′,3′-dihydrosorbicillin (**49**) potentially inhibited α-glucosidase activity [38].

A new derivative of mycophenolic acid named penicacid D (**50**) was isolated from *Penicillium* sp. SCSIO sof101 (the South China Sea). Compound **50** exhibited weak activity against *E. coli* and *Acinetobacter baumannii* [39].

Purpuride D (**51**), a new analogue of drimane-type sesquiterpene lactones conjugated with N-acetyl-l-valine, was isolated from *Penicillum* sp. ZZ1283 (Karachi, Pakistan, the Arabian Sea). Purpuride D showed activities in inhibiting the growth of MRSA, *E. coli* and *Candida albicans* [40].

Four verrucosidin derivatives, penicyrone (**52**), norpenicyrone (**53**), methyl norpenicyrone (**54**), and methyl penicyrone (**55**) were isolated from the hydrothermal vent sulfur-derived fungus *Penicillium* sp. Y-50-10 (Kueishantao, Taiwan, the East China Sea). Compounds **52–55** showed moderate activity against *Bacillus subtilis* [41].

9-Dehydroxysargassopenilline A (**56**) and 1,2-didehydropeaurantiogriseol E (**57**) were isolated from *Penicillium cyclopium* SD-413 (the East China Sea). Compounds inhibited growth of some pathogenic bacteria including *Escherichia coli*, *E. ictaluri*, *Edwardsiella tarda*, *Micrococcus luteus*, *Vibrio anguillarum*, and *V. harveyi* [42].

Tyrosol (**58**) from *Penicillium chrysogenum* DXY-1 (Taiwan Strait) was reported as a potential inhibitor of the quorum sensing (QS) systems to solve the looming crisis of bacterial resistance. The compound significantly decreased QS-regulated violacein production in *Chromobacterium violaceum* CV026 and QS-regulated pyocyanin production, elastase activity, and proteolytic activity in *Pseudomonas aeruginosa* PA01. Moreover, tyrosol inhibited biofilm formation in *P. aeruginosa* PA01 without having any effect on bacterial growth [43].

Penijanthines C (**59**) and D (**60**) as well as penijanthoids A (**61**) and B (**62**), products of *Penicillium janthinellum* (the Bohai Sea) metabolism, displayed significant activity against three pathogenic *Vibrio* spp. [44].

Polycyclic quinazoline alkaloid, thielaviazoline was obtained as a result of the microbial transformation of anthranilic acid by *Thielavia* sp. (Gomso Bay, South Korea, the Yellow Sea). Compound **63** displayed activity against MRSA and MDRSA. Moreover, compound **63** showed potent DPPH radical-scavenging activity [45].

Two new cytochalasans, 16α-methylaspochalasin J (**64**) and 16-hydroxymethylaspergillin PZ (**65**), were isolated from *Westerdykella dispersa* (Guangdong province, China, the South China Sea). Compound **64** exhibited moderate antibacterial activity against *Bacillus subtilis*, while compound **65** was active against *Proteus vulgaris* and *Enterobacter aerogenes* [46].

The salicylaldehyde derivative **66**, isolated from *Zopfiella marina* BCC 18240 (or NBRC 30420, coast of Taiwan), showed moderate activity against *Mycobacterium tuberculosis* H37Ra and *Bacillus cereus* [47].

## 4. Antifungal Activity

Antifungal activity is not rare for metabolites of marine sediment-derived fungi. Presumably, these compounds play a key role in interspecies relationships within fungal communities. Moreover, metabolites that inhibit the growth of aquatic and plant fungal pathogens are of particular importance from a practical point of view.

**Figure 6:**
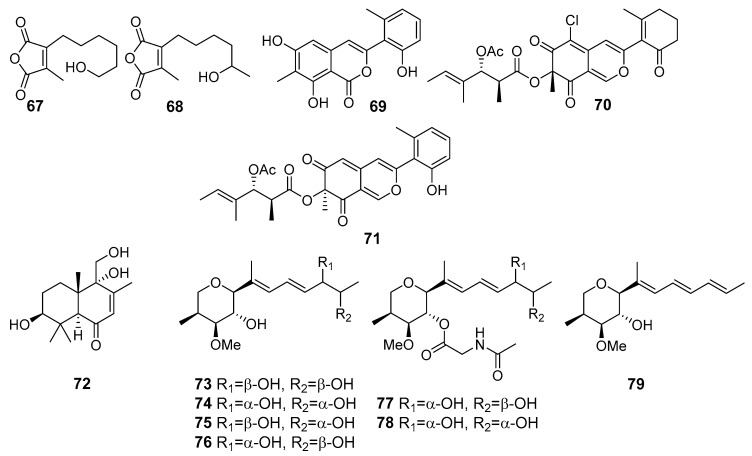
The antifungal metabolites of marine sediment-derived fungi.

Two new furandione derivatives, asperfurandiones A (**67**) and B (**68**) (Figure 6), were isolated from *Aspergillus versicolor* (Dongji Island, China, the East China Sea). Both these metabolites showed moderate activity against *Gaeumannomyces graminis*, *Cryptococcus neoformans*, and *Candida albicans* [48]. 

Novel azaphilone derivatives, named pleosporalone A (**69**), pleosporalones B (**70**) and C (**71**), and pleosporalones E–H, along with three known analogues, were obtained from *Pleosporales* sp. CF09-1 (the Bohai Sea). Pleosporalone A (**69**) showed strong activity against plant pathogenic fungi *Botrytis cinerea*, *Rhizopus oryzae* and *Phytophthora capsici.* Pleosporalone B (**70**) exhibited potent activities against the fungi *Alternaria brassicicola* and *Fusarium oxysporum*. Additionally, pleosporalone C (**71**) displayed significant activity against the fungus *Botryosphaeria dothidea* [49,50].

Later, a large number of drimane sesquiterpenoids were isolated from this fungal strain and one of them, ustusol A (**72**), exhibited activity against a panel of plant pathogenic fungi, including *Thielaviopsis paradoxa*, *Pestalotia calabae*, and *Gloeosporium musarum* [51].

Six new alkenylated tetrahydropyran derivatives belonged to polyketides, designated as (12R,13R)-dihydroxylanomycinol (**73**), (12S,13S)-dihydroxylanomycinol (**74**), (12R,13S)-dihydroxylanomycinol (**75**) and (12S,13R)-dihydroxylanomycinol (**76**), (12S,13R)-N-acetyl-dihydroxylanomycin (**77**) and (12S,13S)-N-acetyl-dihydroxylanomycin (**78**), together with one related known compound lanomycinol (**79**), were isolated from *Westerdykella dispersa* (the South China Sea). Compounds **73**–**79** exhibited moderate antifungal activities selectively against agricultural pathogenic fungi *Rhizoctorzia solani*, *Verticillium dahliae*, *Helminthosporium maydis*, *Fusarium tricinctum, F. oxysporum*, *Botryosphaeria dothidea*, and *Alternaria fragriae* [52].

## 5. Plankton Toxicity

The toxicity of metabolites from marine fungi for phyto- and zooplankton organisms is also important for relationships of fungi in marine ecosystems. Nevertheless, the biological activity of compounds from marine sediment-derived fungi is reported as exceedingly rare.

**Figure 7:**
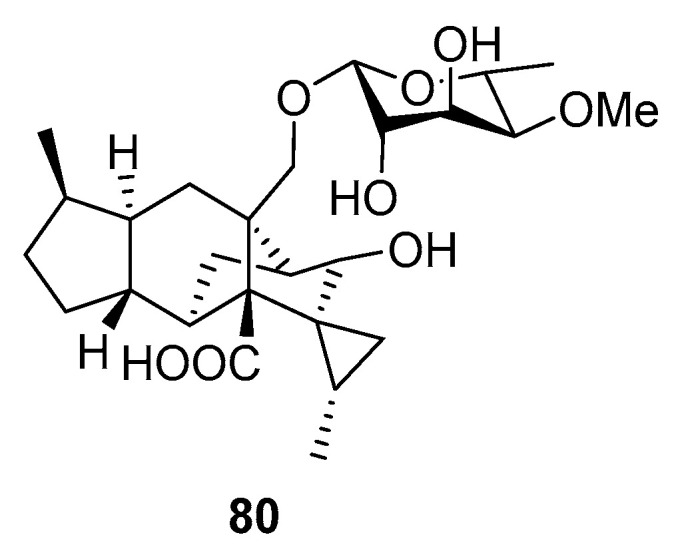
The plankton-toxic metabolites of marine sediment-derived fungi.

A new sordarin derivative, trichosordarin A (**80**), with a unique norditerpene aglycone, was isolated from *Trichoderma harzianum* R5 (the Bohai Sea). Compound **80** (Figure 7) was toxic to *Artemia salina*, but it appeared to only weakly inhibit *Amphidinium carterae* and *Phaeocystis globosa* [53].

## 6. Cytotoxic Activity

According to the Global Burden of Disease Study project data, total cancers resulted in 23.6 million incident cases, 10 million deaths, and 250 million disability-adjusted life years (DALYs) in 204 countries in 2019. Total cancers were the second-leading cause of death globally in 2019 and this trend has continued since 1990 [54].

As a result, the search for leader antitumor molecules among the metabolites of marine fungi is one of the stable modern trends in chemistry of natural compounds [55,56].

**Figure 8:**
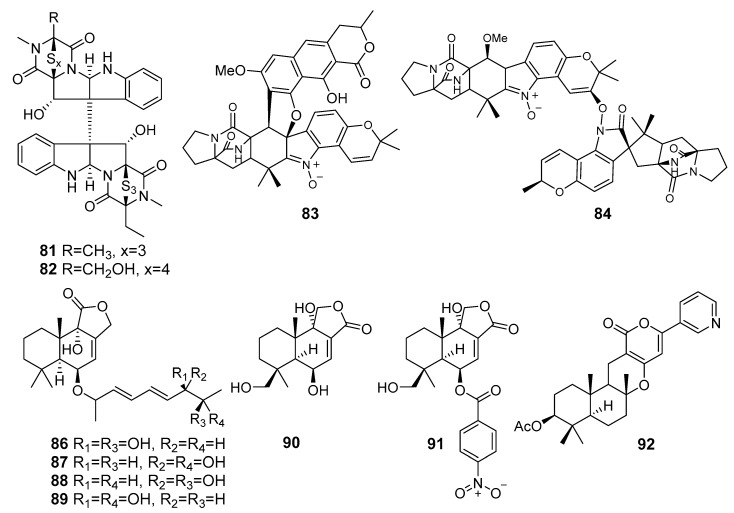
The cytotoxic metabolites (**81**–**92**) of marine sediment-derived fungi.

Two new epipolythiodiketopiperazines, named chetracins E (**81**) and F (**82**) (Figure 8) were isolated from the fungus *Acrostalagmus luteoalbus* HDN13-530 (Liaodong Bay, the Bohai Sea). All the compounds exhibited strong cytotoxicity against the five cancer cell lines. The computational docking indicated that compounds **81** and **82** could bind to the C-terminal of heat shock protein 90 (Hsp90), which was in line with the experimental observation of decreases in levels and active forms of Hsp90 client proteins [57].

Waikikiamide A (**83**), 2,5-diketopiperazine−polyketide hybrid, and waikikiamide C (**84**), an unprecedented 2,5-diketopiperazine dimer with an N−O−C bridge, were isolated from *Aspergillus* sp. FM242 (Waikiki beach, Oahu, Honolulu, Hawaii). These showed strong antiproliferative activity against human fibrosarcoma HT1080 line, human prostatic tumor PC3 line, an immortalized T lymphocyte Jurkat line, and human ovarian cancer A2780 cell lines [58].

Asperienes A–D (**86**–**89**), C-6′/C-7′ epimeric drimane sesquiterpene esters, were isolated from *Aspergillus flavus* CF13-11 (the Bohai Sea). Compounds **86**–**89** displayed potent activities towards HeLa, MCF-7, MGC-803, and A549 cell lines [59].

Two new drimane sesquiterpenes together with two known derivatives were isolated from *Aspergillus flocculosus* (Nha Trang Bay, the South China Sea). Compound **90** and its known nitrobenzoyl ester **91** exhibited cytotoxic activity toward human prostate cancer 22Rv1, human breast cancer MCF-7, and murine neuroblastoma Neuro-2a cells [60].

Two new β-bergamotane sesquiterpenoids, E-β-trans-5,8,11-trihydroxybergamot-9-ene and β-trans-2β,5,15-trihydroxybergamot-10-ene, with three known terpenoids, were isolated from *Aspergillus fumigatus* YK-7 (Yingkou, the Bohai Sea). Known pyripyropene E (**92**) exhibited potent activity against the human leukemic monocyte lymphoma U937 cell line [61].

**Figure 9:**
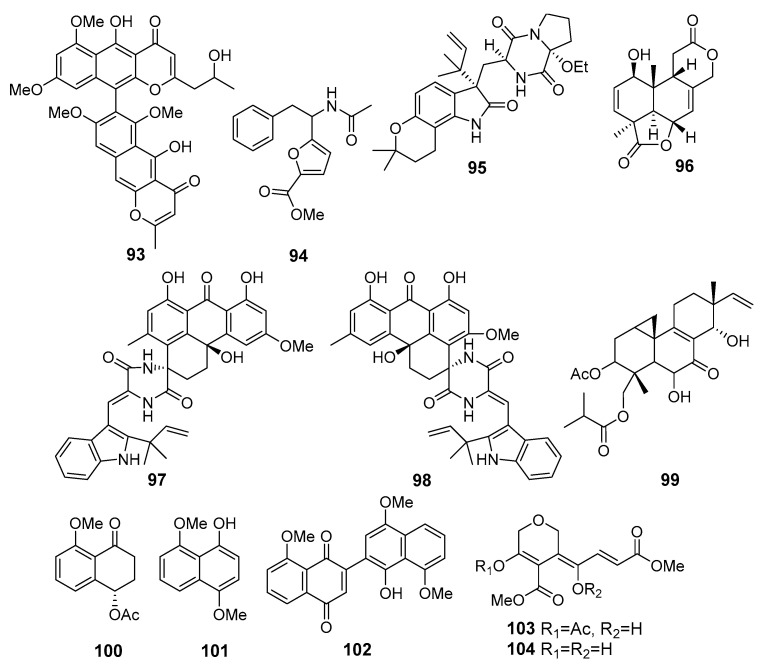
The cytotoxic metabolites (**93**–**104**) of marine sediment-derived fungi.

A new dimeric naphthopyrone, aurasperone H (**93**) (Figure 9), was isolated from *Aspergillus niger* 2HL-M-8 (northeast Brazilian coast, the Atlantic Ocean). Compound **93** exhibited moderate activity against the human lung adenocarcinoma A549 and the human leukemia HL-60 cell lines [62]. Another strain of *Aspergillus niger* from this location produced a new furoic acid derivative (**94**) that exhibited cytotoxicity against HCT-116 cell line [63].

A new alkaloid, 17-O-ethylnotoamide M (**95**), was isolated from a co-culture of marine sediment-derived fungi *Aspergillus sulphureus* KMM 4640 (East Sakhalin shelf, the Sea of Okhotsk) and *Isaria felina* KMM 4639 (the South China Sea, coast of Vietnam). Compound 95 inhibited the colony formation of human prostate cancer 22Rv1 cells [64].

New tetranorlabdane diterpenoid asperolide E (**96**) was isolated from *Aspergillus wentii* SD-310, a producer of antimicrobial isopimaranes **28**–**32**. Asperolide E (**96**) displayed cytotoxic activities against the HeLa, MCF-7, and lung cancer NCI-H446 cell lines [65].

Three pairs of spirocyclic diketopiperazine enantiomers, variecolortins A and B (**97** and **98**), were isolated from *Eurotium* sp. SCSIO F452 (the South China Sea). (+)-**97** showed moderate cytotoxicities against SF-268 and HepG2 cell lines, while those of (+)-**98** were less active [66].

Two new pimarane-type diterpenes, scopararanes, along with five known ones were isolated from *Eutypella* sp. FS46 (the South China Sea). Scopararane I (**99**) showed moderate inhibitory activities against the MCF-7, lung cancer NCI-H460, and SF-268 cell lines [67]. 

Hypoxone A (**100**), 4,8-dimethoxy-1-naphthol (**101**), and 1′-hydroxy-4′,8,8′-trimethoxy[2,2′]binaphthalenyl-1,4-dione (**102**) were isolated from *Hypoxylon rubiginosum* FS521 (the South China Sea). Compound **102** exhibited potent cytotoxic activity against SF-268, MCF-7, HepG-2, and A549 tumor cell lines [68].

Unique prostate cancer-toxic polyketides isariketides A (**103**) and B (**104**) were isolated from marine sediment-derived fungus *Isaria felina* (the South China Sea, coast of Vietnam) [69]. Compound **103** exhibited potent cytotoxicity against several lines of human prostate cancer cells, whereas **104** was inactive. Moreover, authors synthesized an acetate derivative of **103** that showed stronger cytotoxicity in comparison with **103**.

**Figure 10:**
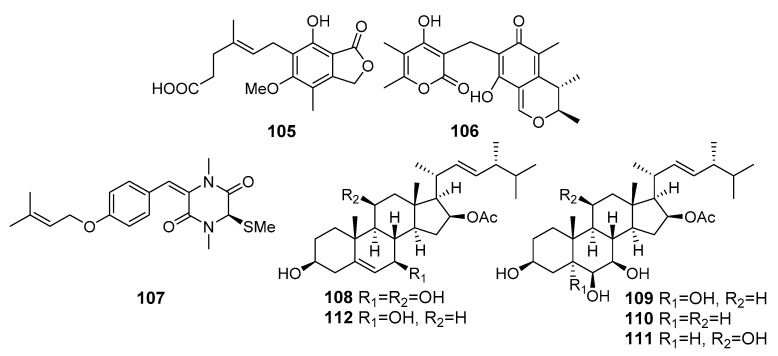
The cytotoxic metabolites (**105**–**112**) of marine sediment-derived fungi.

Eleven compounds including mycophenolic acid and its seven analogues were isolated from *Penicillium brevicompactum* OUCMDZ-4920 (the South China Sea). Mycophenolic acid (**105**) (Figure 10) displayed cytotoxicity against murine leukemia P388, human oral epithelial carcinoma KB, human colorectal cancer HT29, human breast cancer MCF-7, and human lung cancer A549 cells [70].

Dicitrinone D (**106**) as a new polyketide was isolated from *Penicillium citrinum* (southeast coast of China). Dicitrinone D was safe for normal cells, but effectively inhibited the growth of human lung adenocarcinoma SPC-A1 cells [71].

A new diketopiperazine (**107**) was isolated from the Antarctic marine-derived fungus *Penicillium crustosum* HDN153086 (Prydz Bay, the Antarctic Ocean) and exhibited cytotoxicity against K562 cells [72].

New ergostanes, penicisteroids E (**108**), G (**109**) and H (**110**), together with known related penicisteroids A (**111**) and C (**112**) were isolated from *Penicillium granulatum* MCCC 3A00475 (Antarctica). Compounds **108**–**112** showed moderate antiproliferative effects selectively against 12 different cancer cell lines. Compounds **109** and **112,** potent RXRa binders with Kd values of 13.8 µM, could induce apoptosis by a retinoid X receptor (RXR)-α-dependent mechanism by regulating RXRα transcriptional expression and promoting the poly-ADP-ribose polymerase (PARP) cleavage. Moreover, they could inhibit proliferation by cell cycle arrest at the G0/G1 phase [73].

**Figure 11:**
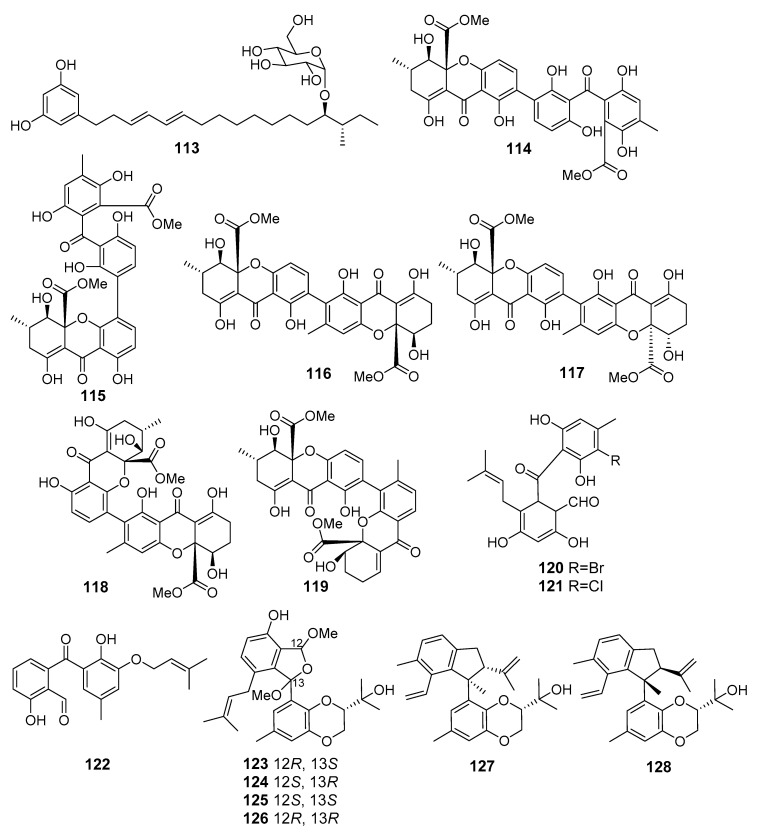
The cytotoxic metabolites (**113**–**128**) of marine sediment-derived fungi.

The fungus *Penicillium janthinellum* (Cu Lao Cham Island, the South China Sea) produced new glycosylated alkylresorcinol resorcinoside A (**113**) (Figure 11) that exhibited cytotoxic activity against the human gastric carcinoma NUGC-3 cell line [74].

New bis-xanthone derivatives, secalonic acids H–M (**114**–**119**) were isolated from *Penicillum oxalicum* fungus (Langqi Island, Taiwan Strait). Secalonic acids H (**114**) and I (**115**) showed a weak cytotoxic effect toward HCT116, KB, and EC9706 cell lines [75], whereas compounds **116**–**119** exhibited moderate cytotoxicity against HeLa, HCT116, MCF-7, Hep-3B, and A549 cells. Moreover secalonic acid J (**116**), isolated from *Penicillium oxalicum* fungus, showed moderate induction of apoptosis in HeLa cells [76].

New halogenated benzophenone derivatives pestalone C (**120**) and pestalone E (**121**), were obtained from *Pestalotiopsis neglecta* (Gagedo Island, the Yellow Sea). Isolated compounds suppressed pancreatic cancer cell line PANC-1 proliferation and induced apoptosis. An in silico study suggested that benzophenone derivatives could potentially inhibit MEK activity by binding to the allosteric pocket in MEK [77].

The marine fungus *Phomopsis lithocarpus* FS508 (the Indian Ocean) produced a new benzophenone derivative tenellone H (**122**) that exhibited strong cytotoxic activity against HepG-2 and A549 cell lines [78]. Later, lithocarols A–D (**123**–**126**) possessing a novel highly-oxygenated isobenzofuran core [79] and lithocarpinols A (**127**) and B (**128**), a pair of tenellone diastereoisomers with novel fused skeleton [80], were isolated from this fungal strain. Compounds **123**–**127** displayed a moderate growth inhibitory effect against HepG-2, MCF-7, SF-268, and A549 human tumor cell lines. Interestingly, lithocarpinol B (**128**) was twice less cytotoxic than its diastereomer **127**.

**Figure 12:**
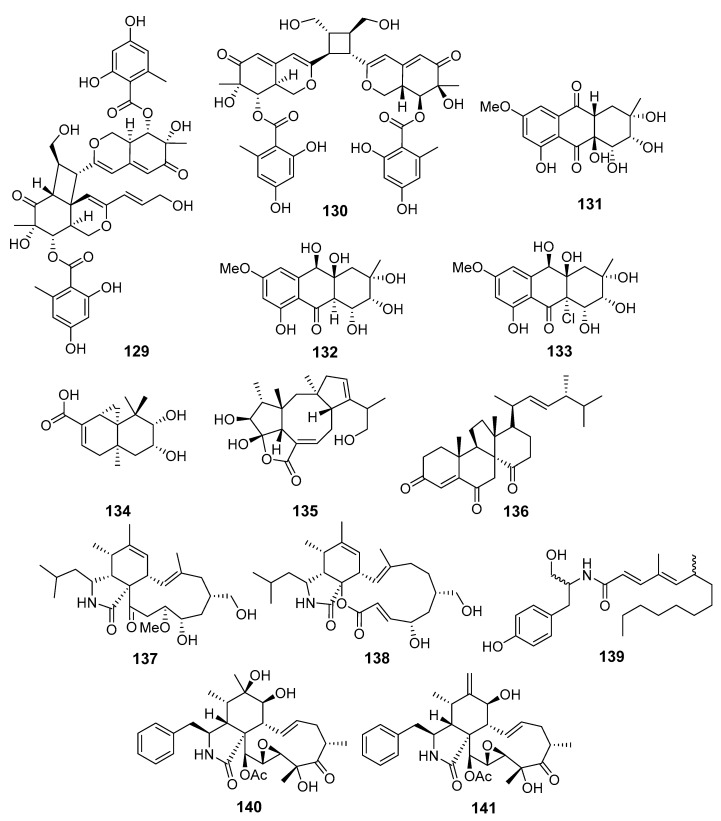
The cytotoxic metabolites (**129**–**141**) of marine sediment-derived fungi.

Dipleosporalones A (**129**) and B (**130**), two new [2+2] azaphilone dimers (Figure 12), were obtained from *Pleosporales* sp. (coast of Huanghua, the Bohai Sea). Dipleosporalone A (**129**) possessed an unprecedented skeleton with an uncommon 6/4/6 ring system. Compounds **129** and **130** showed strong activity against human breast cancer MDA-MB-231 and MCF-7, human gastric cancer MGC-803, cervical cancer HeLa, and human lung epithelial carcinoma A549 cell lines [81].

New anthraquinone derivatives, auxarthrols D–H, together with several known related compounds were obtained from fungus *Sporendonema casei* (Zhangzi Island, the Yellow Sea). Two of them, auxarthrols D (**131**) and F (**132**), showed moderate activities against a few human cancer cell lines. In addition, a weak activity against *Mycobacterium phlei*, *Bacillus subtilis*, *Vibrio parahemolyticus* and *Pseudomonas aeruginosa* was observed for known altersolanol B (**133**) [82].

A new sesquiterpenoid 9,10-diolhinokiic acid (**134**) and a new diterpenoid roussoellol C (**135**), together with known dankasterone (**136**), were isolated from *Talaromyces purpurogenus* (coast of Qinghuangdao, the Bohai Sea). Compound **135** and **136** showed moderate activity toward MCF-7 and HL-60, respectively, whereas **134** possessed only weak cytotoxicity against HL-60 and A549 cells [83].

A number of new cytochalasins together with known analogs and a new tyrosine-derived alkaloid were produced by the fungus *Westerdykella dispersa* (Guangzhou, China, the South China Sea). Some of them, new 19-methoxy-19,20-dihydrophomacin C (**137**), alkaloid gymnastatin Z (**138**), and known phomacin B (**139**), exhibited moderate activities against MCF-7, HepG2, A549, HT-29, and SGC-7901 human cancer cell lines [84]. One more cytochalasin derivative, named cytochalasin P1 (**140**), together with known analog **141**, were isolated from *Xylaria* sp. SOF11 (the South China Sea). Compounds **140** and **141** showed significant cytotoxicity against human breast cancer MCF-7 and human glioblastoma SF-268 cells [85].

## 7. Anti-Inflammatory Activity

Acute and chronic inflammation are the types of cellular response on foreign agent intervention, shock or injury, and hypersensitivity. Inflammation is a necessary response to maintain normal homeostasis in an organism that has been infected or injured. However, prolonged inflammation can cause serious cell and molecule damage which results in different diseases. Normally functioning cells maintain a balance between proinflammatory and anti-inflammatory mediators, and in the event of any action, this balance shifts. The production of proinflammatory mediators during inflammation is promoted by macrophages which include tumor necrosis factor (TNF-a), various interleukins, prostaglandins (PGs), nitric oxide (NO), and reactive oxygen species (ROS) [86]. An increase in the production of these pro-inflammatory mediators is observed in bacterial lipopolysaccharide (LPS)-treated macrophage or microglia cells which are used as inflammation cell models in the search for anti-inflammatory drug candidates. A recent review of anti-inflammatory substances from marine fungi covered the 130 compounds isolated between 2000 and 2018, but did not focus on the source of the fungi [87].

**Figure 13:**
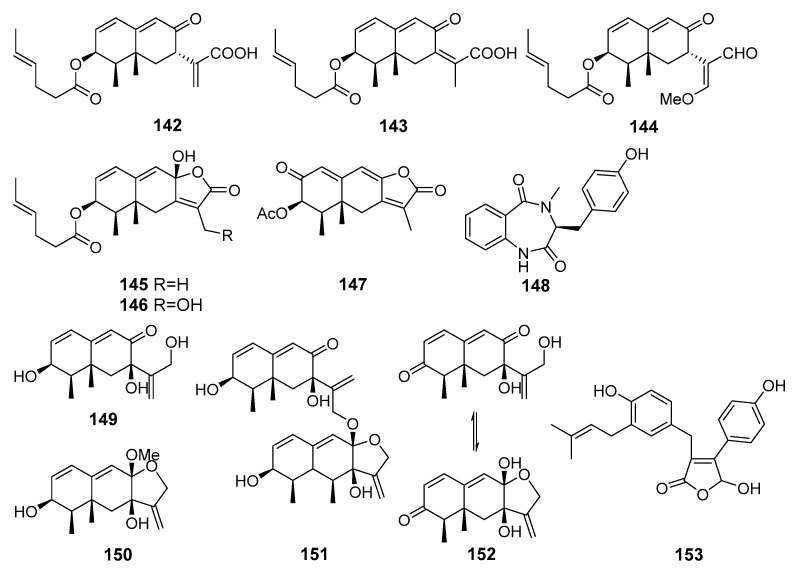
The anti-inflammatory metabolites (**142**–**153**) of marine sediment-derived fungi.

New eremophilane-type sesquiterpenoids acremeremophilanes A-O, together with known analogues, were isolated from *Acremonium* sp. (south part of the Atlantic Ocean). Acremeremophilanes B–F (**142**–**146**) and N (**147**) (Figure 13) exhibited significant inhibitory effects toward NO production in LPS-treated RAW 264.7 macrophage cells [88].

A novel cyclic dipeptide, 14-hydroxy-cyclopeptine (**148**), was isolated from *Aspergillus* sp. SCSIOW2 (the South China Sea). Compound **148** inhibited NO production in LPS and recombinant mouse interferon-γ -activated macrophage RAW 264.7 cells [89]. Moreover, three new eremophilane-type sesquiterpenes, dihydrobipolaroxin B (**150**), dihydrobipolaroxin C (**151**), and dihydrobipolaroxin D (**152**), along with one known analogue, dihydrobipolaroxin (**149**), were isolated from this fungus treated with a combination of histone deacetylase inhibitor (suberohydroxamic acid) and DNA methyltransferase inhibitor (5-azacytidine). Sesquiterpenes **149**–**152** were not produced by the untreated fungal culture. All four compounds exhibited moderate NO inhibitory activities without cytotoxic effects [90].

Three new compounds with novel open-ring butenolide skeletons were isolated from *Aspergillus terreus* Y10 (coastal area of Hainan, the South China Sea). One of them, asperteretal F (**153**) was found to dose-dependently inhibit tumor necrosis factor (TNF-α) generation [91].

**Figure 14:**
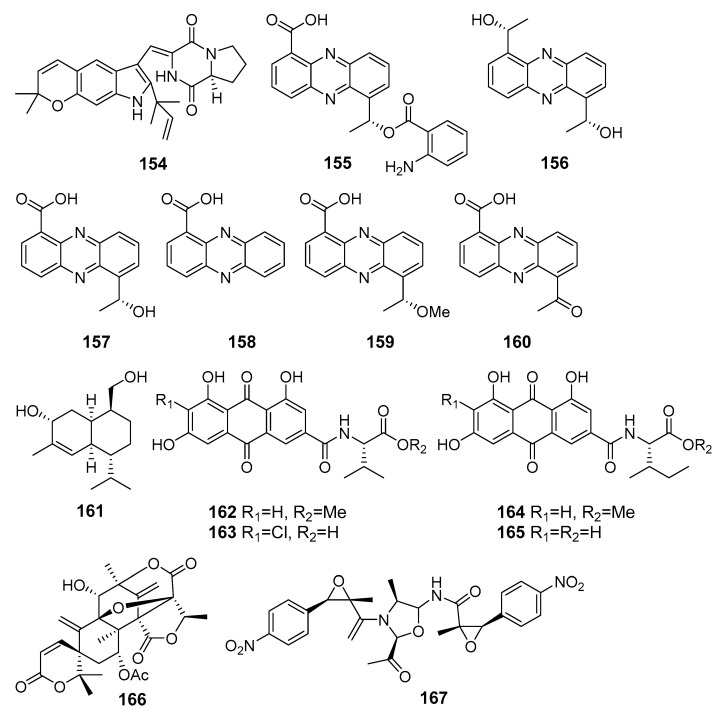
The anti-inflammatory metabolites (**154**–**167**) of marine sediment-derived fungi.

Asperversiamide G (**154**) (Figure 14), possessing an unusual pyrano[3,2-f]indole unit, was isolated from *Aspergillus versicolor* (the South China Sea) and exhibited a potent inhibitory effect against iNO synthase [92].

New phenazine derivatives **155** and **156**, along with known saphenic acid derivatives **157**–**160**, were isolated from a yeast-like fungus *Cystobasidium larynigs* (the Indian Ocean). All isolated compounds **155**–**160** showed a nitric oxide (NO) production inhibitory effect in LPS-induced murine macrophage RAW 264.7 cells [93].

A new cadinane sesquiterpene khusinol B (**161**) isolated from fungus *Graphostroma* sp. MCCC 3A00421 (the Atlantic Ocean) exhibited significant inhibition of nitric oxide (NO) production in LPS-induced RAW 264.7 macrophages [94].

A marine-derived fungus *Penicillium* sp. SCSIO sof101, in addition to antimicrobial polyketide **50**, produced emodacidamides A, C, D, and E (**162**–**165**), featuring anthraquinone-amino acid conjugates. Emodacidamides A (**162**), C (**163**), D (**164**), and E (**165**) inhibited interleukin-2 secretion from Jurkat cells [95].

New meroterpenoid 7-acetoxydehydroaustinol (**166**) isolated from *Penicillium* sp. F-5497 (Busan, South Korea, Korean Strait) weakly suppressed NO overproduction in LPS-challenged BV2 microglial cells [96].

Three dimeric nitrophenyl trans-epoxyamides, chrysamides A–C, were obtained from *Penicillium chrysogenum* SCSIO41001 (the Indian Ocean). Chrysamide C (**167**) suppressed the production of proinflammatory cytokine interleukin-17 [97].

**Figure 15:**
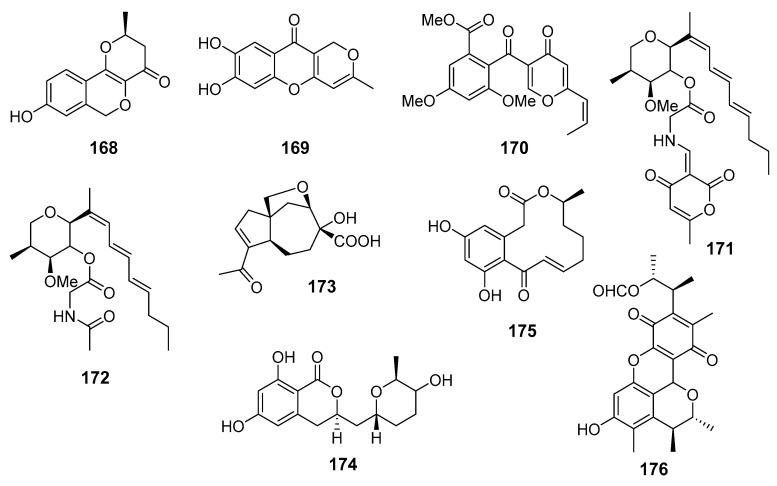
The anti-inflammatory metabolites (**168**–**176**) of marine sediment-derived fungi.

Several known polyketide metabolites, neuchromenin (**168**), myxotrichin C (**169**), and deoxyfunicone (**170**), were isolated from fungal strain *Penicillium glabrum* SF-7123 (the Ross Sea, Antarctica). Compounds **168**, **169**, and **170** (Figure 15) possessed inhibitory activity against production of NO and prostaglandin E2 in LPS-stimulated BV2 microglial and RAW264.7 macrophage cells. The anti-inflammatory effects of **168** were associated with a suppressive effect on iNO synthase and cyclooxygenase-2 activities [98].

Restricticin B (**171**), a new compound containing a triene, a tetrahydropyran ring, and glycine ester functionalities, together with known N-acetyl restricticin (**172**), were obtained from fungus *Penicillium janthinellum* (Cu Lao Cham Island, the South China Sea). Isolated compounds exhibited anti-neuroinflammatory effects in LPS-induced BV-2 microglia cells by suppressing the production of pro-inflammatory mediators [99].

New carotane sesquiterpenoid piltunine E (**173**) together with several known compounds were isolated from fungus *Penicillium piltunense* KMM 4668 (Sakhalin Island, the Sea of Okhotsk). New piltunine E (**173**) and known 5′-hydroxyasperentin (**174**) significantly downregulated ROS production in LPS-stimulated murine peritoneal macrophages. Moreover, **174** decreased NO production in these cells [100].

A number of new and known curvularin-type macrolides were isolated from fungus *Penicillium sumatrense* (the Indian Ocean). Only known dehydrocurvularin (**175**) showed significant inhibition activity towards LPS-induced nitric oxide production in RAW 264.7 macrophages [101].

A known polyketide metabolite citrinin H1 (**176**) was isolated from fungus *Penicillium* sp. SF-5629 (Ulgin, South Korea, the Sea of Japan). Citrinin H1 (**176**) inhibited NO and prostaglandin E2 production in lipopolysaccharide (LPS)-stimulated BV2 microglia cells. Moreover, it was found to suppress cyclooxygenase-2 gene expression and the phosphorylation of inhibitor kappa B-α, to interrupt the nuclear translocation of nuclear factor kappa B, and to decrease the activation of p38 mitogen-activated protein kinase [102].

## 8. Radical Scavenging and Antioxidant Activities

Imbalance between the pro-oxidant and the antioxidant components of homeostatic systems, i.e., oxidative stress, results in different cellular pathological processes and diseases including diabetes, neurodegeneration, cardiovascular diseases, and others. Primary antioxidants scavenge radical species, converting them into more stable radicals or non-radical species. Secondary antioxidants quench singlet oxygen, decompose peroxides, chelate pro-oxidative metal ions, and inhibit oxidative enzymes. Moreover, four reactivity-based lines of defense have been identified: preventative antioxidants, radical scavengers, repair antioxidants, and those relying on adaptation mechanisms [103].

**Figure 16:**
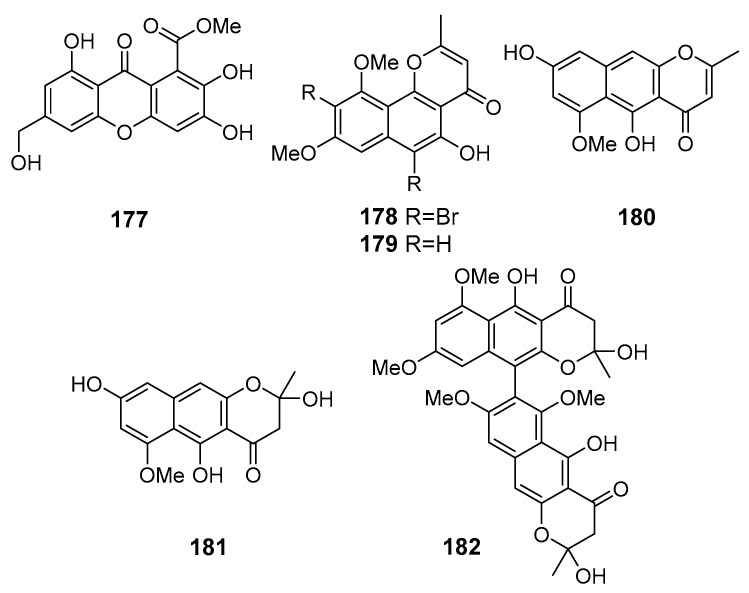
The antioxidant metabolites (**177**–**182**) of marine sediment-derived fungi.

A new chromone derivative arthone C (**177**) (Figure 16) was isolated from *Arthrinium* sp. UJNMF0008 (the South China Sea) and exhibited potent DPPH- and ABTS-radical scavenging activities [104].

A new brominated naphthopyranone 6,9-dibromoflavasperone (**178**) and three known naphtho-c-pyranone monomers, flavasperone (**179**), TMC-256A1 (**180**), and fonsecin (**181**), and one naphtho-c-pyranone dimer aurasperone B (**182**), were isolated from *Aspergillus niger* (Suncheon Bay, South Korea, Korean Strait) fermented with metal bromides (NaBr and CaBr_2_). Compounds **178**–**182** displayed potent DPPH-radical scavenging activity [105].

**Figure 17:**
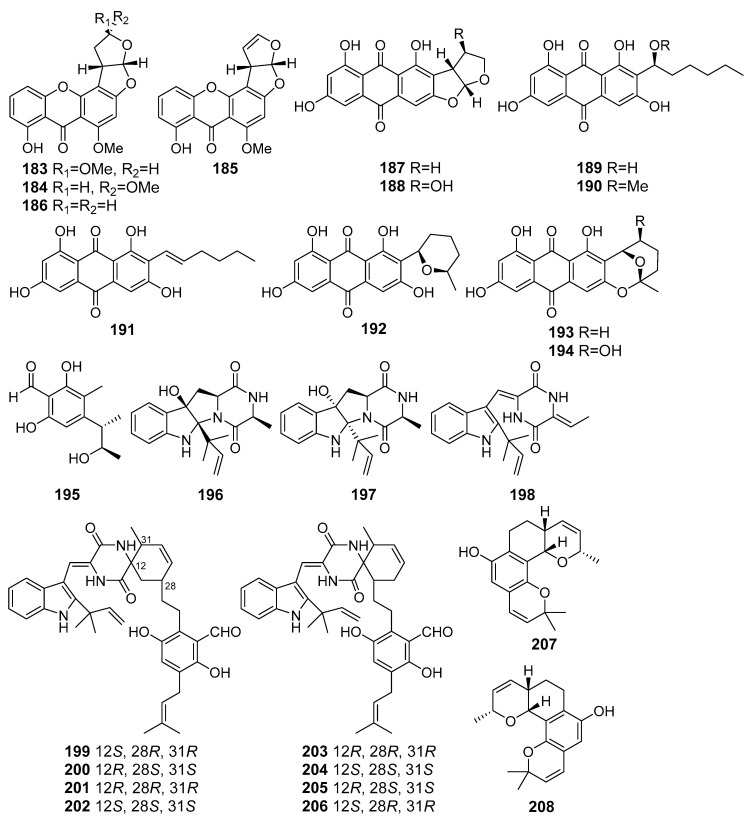
The antioxidant metabolites (**183**–**208**) of marine sediment-derived fungi.

A number of xanthones and anthraquinones (**183**–**194**), including a new xanthone, oxisterigmatocystin D (**183**), were isolated from *Aspergillus versicolor* (the South China Sea). The isolated xanthones and anthraquinones (Figure 17) showed moderate antioxidant activities in trolox-equivalent antioxidant capacity (TEAC) assay. A Nrf2-dependent luciferase reporter gene assay revealed that compound **188**, averantin (**189**), averythrin (**191**), and nidurufin (**194**) potentially activated the expression of a Nrf2-regulated gene [106].

A new citrinin derivative, cladosporin D (**195**), isolated from fungus *Cladosporium* sp. SCSIO z015 (Okinawa Trough, the East China Sea), showed significant DPPH radical scavenging activity [107].

Three new prenylated indole 2,5-diketopiperazine alkaloids (**196**–**198**) were isolated from fungus *Eurotium* sp. SCSIO F452 (the South China Sea). Compound **198** showed significant DPPH-radical scavenging activities. Compounds **196** and **197** exhibited moderate antioxidative activities [108]. Further investigation of this fungal strain led to the isolation of several new spirocyclic indolediketopiperazine alkaloid enantiomers, eurotinoids A–C (**199**–**204**), as well as a known biogenetically related racemate dihydrocryptoechinulin D (**205**/**206**). The isolated compounds showed significant DPPH-radical scavenging activities. In addition, (+)-dihydrocryptoechinulin D (**205**) showed moderate cytotoxicity against SF-268 and HepG2 cell lines [109].

One more study of this fungus, carried out using scaling-up cultivation, resulted in obtaining new salicylaldehyde derivative enantiomers, (+) and (–)-euroticins B (**207** and **208**). The isolated compounds represent the first example of 6/6/6/6 tetracyclic salicylaldehyde derivatives and exhibited remarkable antioxidative activities [110].

**Figure 18:**
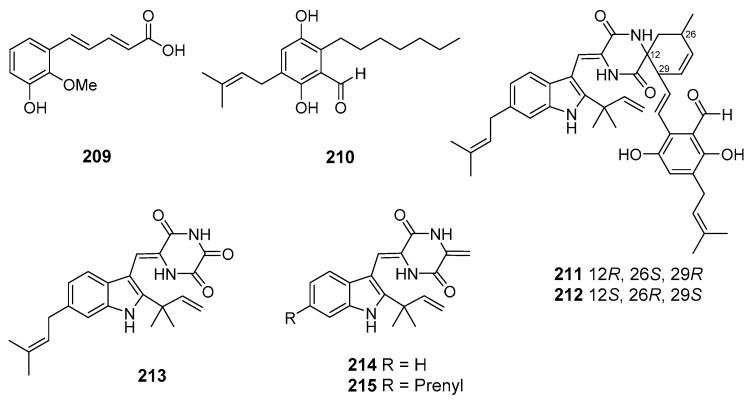
The neuroprotective metabolites of marine sediment-derived fungi.

A new auroglaucin-derived compound, niveoglaucin A (**209**), together with known related compound flavoglaucin (**210**), as well as indolediketopiperazine alkaloids (±)-cryptoechinulines B (**211**–**212**), neoechinulin (**213**), and neoechinulines B–C (**214**–**215**) (Figure 18), were isolated from fungus *Aspergillus niveoglaucus* (Nha Trang Bay, the South China Sea). All these compounds suppressed the hyperproduction of ROS in toxins-treated murine neuroblastoma Neuro-2a cells. Moreover, niveoglaucin A (**209**) and flavoglaucin (**210**) increased viability of 6-hydroxydopamine (6-OHDA)-treated Neuro-2a cells. (+)-Cryptoechinuline B (**211**) exhibited neuroprotective activity on 6-OHDA-, paraquat-, and rotenone-induced the cells. (−)-Cryptoechinuline B (**212**) and neoechinulin C (**215**) protected the Neuro-2a cells against paraquat toxicity. Neoechinulin B (**214**) exhibited cytoprotective activity against rotenone toxicity, and neoechinulin (**213**) showed activity against 6-OHDA [111,112].

## 9. Influence on Protein Activity and Expression

Different proteins are molecular targets in the search for promising drug molecules.

Researchers of anticancer candidates are focusing on proteins involved in cell cycle control and tumor growth as well as metastasis, apoptosis, and others. Bromodomain-containing protein (BRD4) is the most extensively and thoroughly studied member of bromodomain and the extra-terminal domain family. BRD4 plays an important role in cell cycle control and can affect the processes of cell proliferation, apoptosis, and transcription. In addition to its role in tumors, BRD4 also plays an important role in inflammation, cardiovascular diseases, and viral infections [113]. Glycogen synthase kinase-3 (Gsk-3) is a conserved serine/threonine kinase that mainly participates in cell proliferation, development, stress, and inflammation in humans. Accumulating evidence has suggested that GSK 3 beta is correlated with tumorigenesis and progression. However, GSK 3 beta is controversial due to its bifacial roles of tumor suppression and activation. In addition, overexpression of GSK 3 beta is involved in tumor growth, and it contributes to cell sensitivity to chemotherapy [114]. Moreover, it was reported that Gsk-3 protein can be activated in SARS-CoV-2 viral infected cells which results in excessive oxidative stress via degradation of the nuclear factor erythroid 2-related factor (Nrf2) protein. Activated Gsk-3 also modulates CREB-DNA activity, phosphorylates NF-kappa B, and degrades beta-catenin, thus provoking systemic inflammation [115].

The prevalence of diabetes mellitus, especially type 2, has increased significantly by nearly 40% globally in the past 10 years (http://www.healthdata.org/results/gbd_summaries/2019/diabetes-mellitus-level-3-cause, accessed on 25 December 2020). Various enzymes are considered as therapeutic targets for treating this socially significant disease. Alpha-glucosidase is a family of enzymes originating from the pancreas which play a role in the anabolism of 80–90% of carbohydrates consumed into glucose. Inhibition of these enzymes helps to prevent postprandial hyperglycemia and the formation of glycated end products [116]. Protein-tyrosine phosphatase 1B (PTP1B) negatively regulates insulin signaling pathways and plays an important role in type 2 diabetes mellitus (T2DM), as its overexpression may induce insulin resistance [117].

The acetylcholinesterase enzyme (AChE) is the key enzyme in the hydrolysis of the neurotransmitter acetylcholine and is the target of most of the clinically used drugs for the treatment of Alzheimer’s disease [118].

Sterol O-acyltransferase (SOAT) is an endoplasmic reticulum resident, multitrans membrane enzyme that belongs to the membrane-bound O-acyltransferase (MBOAT) family. It catalyzes the esterification of cholesterol to generate cholesteryl esters for cholesterol storage. In addition to cholesterol, SOAT can use multiple sterols as substrates and activators. Because of its functional importance, SOAT is a potential drug target for Alzheimer’s disease, atherosclerosis, and several types of cancers [119].

The heat shock proteins (Hsps), also named “housekeeping” proteins, constitute a large family of molecular chaperones. Their functions focus on protein folding and refolding and other mechanisms of cytoprotecting. Overexpression of Hsps enhances tolerance of cells to stress factors and increases its viability but it is good only for non-malignant cells, for example in the case of neurodegenerative diseases. In cancer cells, Hsps overexpression results in its higher resistance to drug and radiation anticancer therapy. Thus, Hsps (especially Hsp70) are molecular targets for anticancer (Hsps inhibitors) [120] and cytoprotective (Hsps enhancers) [121] drugs.

**Figure 19:**
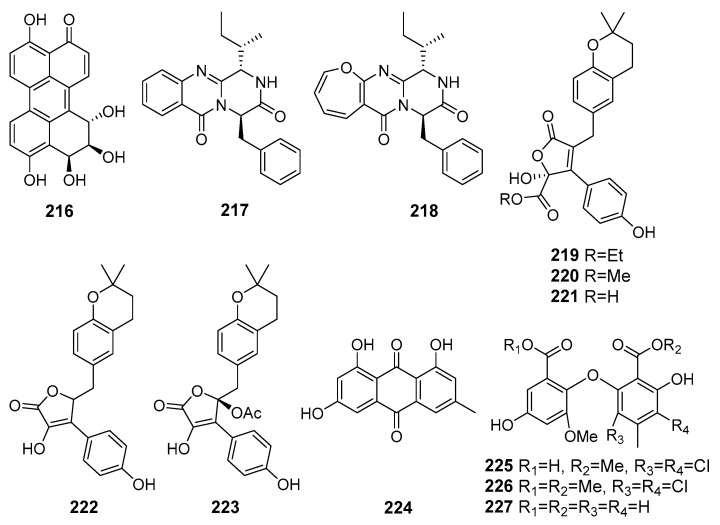
The metabolites (**215**–**227**) of marine sediment-derived fungi with influence on protein activity and expression.

A new perylenequinone (**216**) (Figure 19) isolated from fungus *Alternaria* sp. NH-F6 (the South China Sea) exhibited a potent bromodomain-containing protein BRD4 inhibition [122].

A new diketopiperazine-like compound, designated protuboxepin K (**217**), was isolated together with the known structurally related protuboxepin A (**218**) from *Aspergillus* sp. BFM-0085 (Tokyo Bay, Tokyo, Japan). Compounds **217** and **218** inhibited bone morphogenetic protein (BMP)-induced alkaline phosphatase activity [123].

Three new butenolide derivatives, flavipesolides A–C (**219**–**221**), along with known compounds (**222**–**227**), were isolated from fungus *Aspergillus flavipes* HN4-13 (the Yellow Sea). Known compounds **222**–**224** and **226** were reported as noncompetitive α-glucosidase inhibitors, whereas new flavipesolides A–C (**219**–**221**) and known **225** and **227** were described as mixed α-glucosidase inhibitors [124].

**Figure 20:**
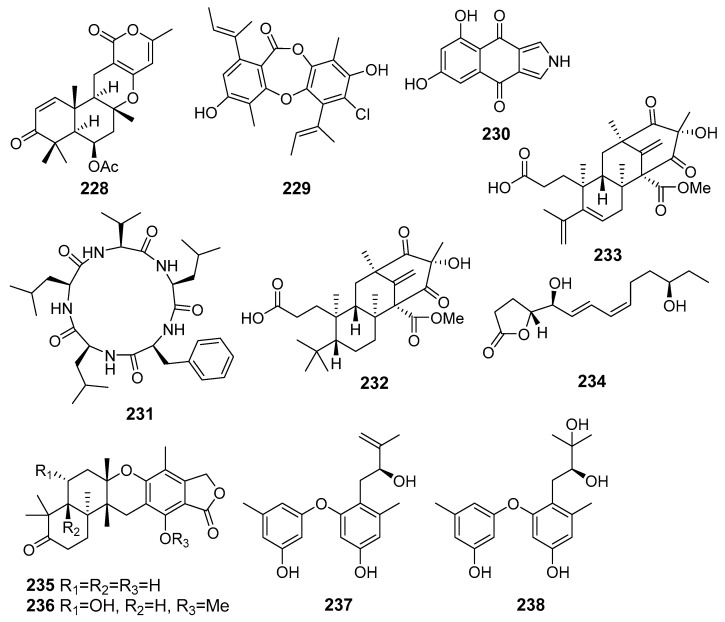
The metabolites (**228**–**238**) of marine sediment-derived fungi with influence on protein activity and expression.

A new merosesquiterpenoid asperversin G (**228**) (Figure 20) obtained from fungus *Aspergillus versicolor* (the South China Sea) exhibited moderate inhibitory activity against the acetylcholinesterase enzyme (AChE) [125].

A new depsidone, named 7-chlorofolipastatin (**229**), was isolated from *Aspergillus ungui* (Suruga Bay, Japan). 7-Chlorofolipastatin inhibited sterol O-acyltransferase (SOAT) 1 and 2 isozymes in cell-based and enzyme assays using SOAT1- and SOAT2-expressing Chinese hamster ovary (CHO) cells [126].

A new isopyrrolonaphthoquinone biscogniauxone (**230**) and cyclo-(l-Phe-l-Leu-l-Val-l-Leu-l-Leu) (**231**) were isolated from *Biscogniauxia mediterranea* (Herodotes Deep, the Mediterranean Sea) and showed inhibitory activity against glycogen synthase kinase (GSK-3β) [127].

New preaustinoid-related meroterpenoid, preaustinoid A6 (**232**) and known berkeleyone C (**233**) were isolated from fungus *Penicillium* sp. SF-5497 (Gijang-gun, Busan, South Korea, Korean Strait) and inhibited PTP1B activity in a dose-dependent manner [128].

A new furanone derivative, butanolide A (**234**), was isolated from the fungus *Penicillium* sp. S-1-18 (Antarctica). Compound **234** showed moderate inhibitory activity against PTP1B [129].

New meroterpenoids, austalides V−X, were isolated from *Penicillium rudallense* (Ga-geo Island, South Korea, the Yellow Sea). Among them, austalides V (**235**) and W (**236**) exhibited potent inhibitory activity on the receptor activator of nuclear factor *κ*B ligand (RANKL)-induced osteoclast differentiation [130].

New diorcinol J (**237**) and known diorcinol B (**238**) was obtained from the EtOAc extract of a co-culture of marine isolates of the fungi *Aspergillus sulphureus* KMM 4640 (the Sea of Okhotsk) and *Isaria felina* KMM 4639 (the South China Sea). The isolated compounds exhibited a weak hemolytic activity and cytotoxicity toward murine Ehrlich carcinoma cells. Moreover, known diorcinol B (**238**) was able to enhance expression of heat shock protein Hsp70 in Ehrlich ascites carcinoma cells [131].

## 10. Other Activities

**Figure 21:**
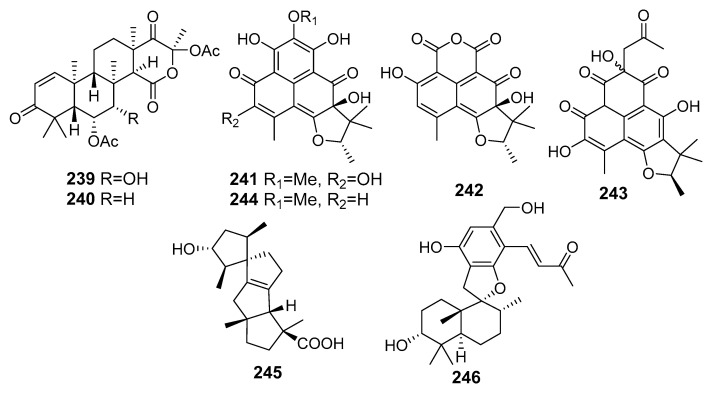
The metabolites of marine sediment-derived fungi with other activities.

Two new sesterterpenoids, terretonins H (**239**) and I (**240**), were isolated from *Aspergillus ustus* KMM 4664 (the Sea of Okhotsk). Compounds (Figure 21) inhibited the ability of spermatozoa to fertilize egg cells of the sea urchin *Strongilocentrotus intermedius* [132].

New phenalenone derivatives *ent*-peniciherqueinone (**241**) and 4-hydroxysclerodin (**242**), along with known compounds (**243**–**244**) of the herqueinone class, were isolated from *Penicillium* sp. (Gagudo, South Korea) [133]. 4-Hydroxysclerodin (**242**) exhibited moderate anti-angiogenetic and anti-inflammatory activities. Compound **243** moderately inhibited NO production in RAW 264.7 cells with an IC_50_ value of 3.2 μM, while the rest of the isolated compounds were inactive (IC_50_ > 20 μM). In the angiogenesis assay, **242** inhibited tube formation in HUVECs with an IC_50_ of 20.9 μM, while *ent*-peniciherqueinone (**241**) and isoherqueinone (**244**) induced adipogenesis through PPAR binding and adiponectin secretion-promoting activity in hBM-MSCs and in a concentration-dependent manner, which was determined by adiponectin secretion-promoting effects with their IC_50_ values of 57.5 μM and 39.7 μM, respectively.

A novel spiro-tetracyclic diterpene, spirograterpene A (**245**), was isolated from fungus *Penicillium granulatum* MCCC 3A00475, that also produced cytotoxic ergostanes **108**–**112**. The isolated compound showed modest antiallergic activity [134].

A new phenylspirodrimane derivative, stachybotrysin (**246**), was isolated from fungus *Stachybotrys* sp. KCB13F013 (Wi Island, South Korea, the Yellow Sea). Stachybotrysin exhibited an inhibitory effect on osteoclast differentiation in bone marrow macrophage cells via suppressing the RANKL-induced activation of p-ERK, p-JNK, p-p38, c-Fos, and NFATc1 [135].

## 11. Concluding Remarks

Marine sediment-derived fungi are exposed to both natural stress factors, to which adaptive mechanisms have already been developed during evolution, and modern challenges, namely the effect of high concentrations of xenobiotics entering sea water because of pollution and settling to the bottom.

Melanization of hyphae is one of the protection mechanisms against changes in osmotic pressure. Thus, adding tricyclazole, a specific inhibitor of the dihydroxynaphthalene (DHN) type of melanin, in *Cirrenalia pygmea* growth medium resulted in producing light-colored hyphae which were highly susceptible to osmotic shock [136].

Changes in secondary metabolite production is a strategy for intracellular osmoregulation. Increasing salinity increased the activity of polyol enzymes such as polyol dehydrogenase and mannitol dehydrogenase and, finally, polyols content. Higher salinity also led to an increase in the amino acid pool size as well as glycogen and sterols. Moreover, higher salinity brought about a decrease in the extent of unsaturation of fatty acids. A hypo-osmotic shock resulted in a decrease in the polyol content [137].

Melanin and its related compounds are able to tolerate marine-derived fungi oxidative stress inducing UV radiation and xenobiotics. The isolation of melanin biosynthesis intermediates from marine-derived fungus and finding their antioxidant and cytoprotective activities confirms the role of them in cell protective machinery [138].

Dihydroxynaphthalene (DHN) melanin pathway is a basic melanin formation in fungi [139] and it is a special case of polyketide pathways which result in a big structural variety of polyketide metabolites [140].

**Figure 22:**
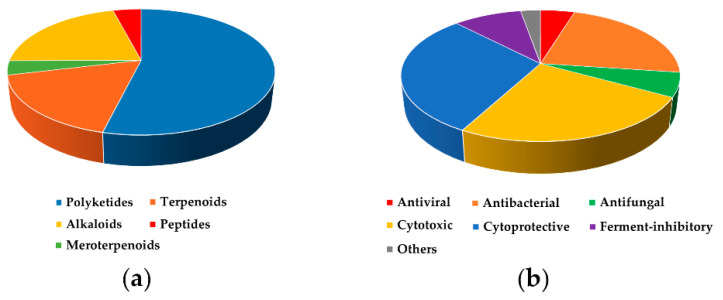
Distribution of bioactive metabolites by (**a**) structural types and (**b**) types of activity.

In total, 246 compounds with various biological activities were reported from 2016 to November 2020 (Appendix A Table A1). In terms of chemical structures, most of them belong to polyketides (54%), and alkaloids (21%) and terpenoids (17%) combined are the second most common. The rarest bioactive compounds are peptides (4%) and meroterpenoids (4%) (Figure 22a).

Antiviral activity against various subtypes of influenza virus and herpes simplex virus 1 has been reported for 12 compounds. For a number of reasons, an investigation of antiviral activity is one of the most difficult bioassays and this is a limiting factor for the discovery of new antiviral drug candidates. Nevertheless, the studying of secondary metabolites from marine sediment-derived fungi for their antiviral activity is promising. The COVID-19 pandemic showed humanity’s vulnerability, but advances in this field, e.g., anti-HIV therapy, offer hope.

Antibacterial activity has been reported for 57 compounds from marine sediment-derived fungi. With the aim of helping to prioritize the research and development of new and effective antibiotic treatments, the WHO recently published a list of bacterial pathogens for which new antibiotics are urgently needed at a global level [141]. Priority I (critical) comprises carbapenem-resistant *Acinetobacter baumannii*, *Pseudomonas aeruginosa*, and members of Enterobacteriaceae, that is, *Klebsiella pneumoniae*, *Escherichia coli*, *Enterobacter* spp., *Serratia* spp., *Proteus* spp., *Providencia* spp., and *Morganella* spp. Representatives of the group Priority II (high) are vancomycin-resistant *Enterococcus faecium* and methicillin- and/or vancomycin-resistant *Staphylococcus aureus*. Penicillin-non-susceptible *Streptococcus pneumoniae*, among others, belongs to Priority III (medium).

In this regard, such marine sediment-derived fungal metabolites as 2-(dimethoxymethyl)-1-hydroxyanthracene-9,10-dione (**26**), emerixanthone E (**33**), emerimicin IV (**34**), penicacid D (**50**), and tyrosol (**58**), which show significant activity against one or more these bacterial pathogens, may be of particular interest for further research.

Cytotoxic activity against various cancer line cells was reported for 62 compounds isolated from marine sediment-derived fungi.

Many natural and semi-synthetic compounds with various antitumor effects are published annually. At first glance, this does not seem to lead to the desired success in new low-molecular-weight drug development. At this time there is only one successful drug candidate for anticancer therapy among marine fungal metabolites. It is plinabulin, a synthetic derivative of phenylahistine (from algicolous *Aspergillus ustus*), that is under Phase II and III of clinical trials in complex therapy of various cancers [142]. From 2005 to 2015, cytotoxic activity was reported for 37% of isolated bioactive fungal metabolites (Introduction section) and from 2016 to 2020 it was reported only for 25% of the compounds (Figure 22b).

More than 70 secondary metabolites with various cytoprotective properties have been isolated from marine sediment-derived fungi from 2016 to now. Anti-inflammatory activity was described for 33 compounds. Radical scavenging and antioxidant activities were reported for 37 compounds and for 7 from them a neuroprotective effect was shown. Moreover, enhancing of Hsp70 expression was reported for one compound.

As was noted in the Introduction, only nine compounds with cytoprotective properties have been identified from 2005 to 2015. Of course, it should be borne in mind that the biological activity could have been studied and published later, but a change in the trend in the study of the biological effects of compounds is clearly visible.

It is obvious that further development of the field of marine fungal metabolites investigation will be determined by improvements of the existing isolation and identification techniques, as well as by the arrival of fundamentally new approaches. Moreover, at present, there is an increasing interest in studies of the fungal metabolome that give perspectives in complex estimation of biosynthetic abilities of fungal strains and the influence of ecological factors on fungal metabolomes. Using gene cluster technologies together with the metabolome approach will allow controlling of the fungal biosynthetic pathways to obtain metabolites with the expected biological properties.

## Appendix A

**Table A1:** Bioactive compounds from marine sediment-derived fungi from 2016 to 2020.

Compound	Fungus	Isolation place	Activity	Ref.
Acremonpeptide A (**1**)	*Acremonium persicinum* SCSIO115	The South China Sea	Antiviral (herpes simplex virus 1)	[15]
Acremonpeptide B (**2**)				
Al(III)—acremonpeptide D (**3**)				
Coniochaetone J (**4**)	*Penicillium* sp. SCSIO Ind16F01	The Indian Ocean	Antiviral (enterovirus EV71), Cytotoxic (K562, MCF-7, SGC7901)	[16]
Epiremisporine B (**5**)			Antiviral (enterovirus EV71, influenza A virus subtype H3N2), Cytotoxic (K562, MCF-7, SGC7901)	
Spiromastilactone D (**6**)	*Spiromastix* sp.	South of The Atlantic Ocean	Antiviral (a panel of influenza A and B viruses)	[17]
Diorcinol K (**7**)	*Aspergillus* sp. CUGB-F046	The Bohai Sea, China	Antibacterial (SA, MRSA)	[19]
Diorcinol D (**8**)				
Diorcinol I (**9**)				
Aspergixanthone H (**11**)	*Aspergillus* sp. ZA-01	The Bohai Sea	Antibacterial *(Micrococcus lysodeikticus)*	[20,21]
Aspergixanthone I (**12**)			Antibacterial *(Vibrio parahemolyticus*, *V. anguillarum*, and *V. alginolyticus)*	
Aspergixanthone G (**10**)				
JG002CPA (**13**)	*Aspergillus allahabadii*	Jeju Island, Korea	Cytotoxic (A-549)	[22]
JG002CPB (**14**)				
FJ120DPA (**15**)	*Aspergillus ochraceopetaliformis*			
FJ120DPB (**16**)				
Fiscpropionates A–D (**17**–**20**)	*Aspergillus fischeri* FS452	Indian Ocean	Antibacterial *(Mycobacterium tuberculosis* protein tyrosine phosphatase B inhibition)	[23]
Helvolinic acid (**21**)	*Aspergillus fumigatus* MF071	The Bohai Sea	Antibacterial (SA, E. *coli*)	[24]
Helvolic acid (**22**)				
Bisvertinolone (**23**)	*Aspergillus protuberus* MUT3638	Hammerfest fiord, The Barents Sea	Antibacterial (SA)	[25]
Versicolorin B (**24**)	*Aspergillus versicolor* MF180151	The Bohai Sea	Antibacterial (SA, MRSA)	[26]
Averufin (**25**)				
2-(Dimethoxymethyl)-1-hydroxyanthracene-9,10-dione (**26**)	*Aspergillus versicolor*	West Pacific Ocean	Antibacterial (MRSA), inhibition of topoisomerase IV and AmpC β-lactamase enzymes activity	[27]
(3*R*,9*S*,12*R*,13*S*,17*S*,18*S*)-2-carbonyl-3-hydroxylemeniveol (**27**)	*Aspergillus versicolor* ZZ761	Shengsi Island, The East China Sea	Antibacterial and antifungal (*E. coli* and *Candida albicans*)	[28]
Aspewentins D–H (**28**–**32**)	*Aspergillus wentii* SD-310	The South China Sea	Antibacterial (*Edwardsiella tarda*, *Micrococcus luteus*, *Pseudomonas aeruginosa*, *Vibrio harveyi*, and *V. parahemolyticus*), antifungal (*Fusarium graminearum)*	[29]
Emerixanthone E (**33**)	*Emericella* sp.	The South China Sea	Antibacterial *(SA*, *E. coli*, *Klebsiella pneumoniae*, *Enterococcus faecalis*, *Acinetobacter baumannii*, *Aeromonas hydrophila)*	[30]
Emerimicin IV (**34**)	*Emericellopsis minima*	Talcahuano Bay, Chile	Bacteriostatic (MRSA, VRE)	[31]
Ethyl 3,5-dimethoxy-2-propionylbenzoate (**35**)	*Engyodontium album*	The Pacific Ocean	Antibacterial (MRSA and *Vibrio vulnificus)*	[32]
Engyodontiumin A (**36**)			Antibacterial (MRSA, *Vibrio vulnificus*, *V. rotiferianus*, and *V. campbellii),* antifungal (*Aspergillus niger*)	[33]
Eutypellazines P–R (**37**–**39**)	*Eutypella* sp. MCCC 3A00281	The South Atlantic Ocean	Antibacterial (SA*,* VRE)	[34]
Eutypellol A (**40**)	*Eutypella scoparia* FS46	The South China Sea	Antibacterial (SA)	[35]
Eutypellol B (**41**)				
2-(2-Hydroxy-4-methylcyclohex-3-enyl)propanoic acid (**42**)				
Oxysporizoline (**43**)	*Fusarium oxysporum*	Suncheon Bay, South Korea, Korea Strait	Antibacterial (MRSA, MDRSA)	[36]
Mycousfuran A (**44**)	*Mycosphaerella* sp.	Donghae-si, Gangwon-do, South Korea, The Japanese Sea	Antibacterial *(Kocuria rhizophila* and SA)	[37]
Mycousfuran B (**45**)				
2-[(5-Methyl-1,4-dioxan-2-yl)methoxy]ethanol (**46**)	*Penicillium* sp. M30	Con Co Island, The South China Sea	Antibacterial *(Enterococcus faecalis)*	[38]
2-[(2R-Hydroxypropanoyl)amino]benzamide (**47**)			Antibacterial *(E. coli)*	
4-Hydroxybenzandehyde (**48**)				
2′,3′-Dihydrosorbicillin (**49**)			Inhibition of α-glucosidase activity	
Penicacid D (**50**)	*Penicillium* sp. SCSIO sof101	The South China Sea	Antibacterial *(E. coli* and *Acinetobacter baumannii)*	[39]
Purpuride D (**51**)	*Penicillum* sp. ZZ1283	Karachi, Pakistan, The Arabian Sea	Antibacterial (MRSA, *E. coli*), antifungal (*Candida albicans)*	[40]
Penicyrone (**52**)	*Penicillium* sp. Y-50-10	Kueishantao, Taiwan, The East China Sea	Antibacterial *(Bacillus subtilis)*	[41]
Norpenicyrone (**53**)				
Methyl norpenicyrone (**54**)				
Methyl penicyrone (**55**)				
9-Dehydroxysargassopenilline A (**56**)	*Penicillium cyclopium* SD-413	The East China Sea	Antibacterial *(E. coli*, *E. ictaluri*, *Edwardsiella tarda*, *Micrococcus luteus*, *Vibrio anguillarum*, and *V. harveyi)*	[42]
1,2-Didehydropeaurantiogriseol E (**57**)				
Tyrosol (**58**)	*Penicillium chrysogenum* DXY-1	Taiwan Strait	Antibacterial (inhibitor of the quorum sensing (QS) system in *Chromobacterium violaceum andPseudomonas aeruginosa,* biofilm formation in *P. aeruginosa*)	[43]
Penijanthines C (**59**)	*Penicillium janthinellum*	The Bohai Sea	Antibacterial *(Vibrio* spp.)	[44]
Penijanthine D (**60**)				
Penijanthoid A (**61**)				
Penijanthoid B (**62**)				
Thielaviazoline (**63**)	*Thielavia* sp.	Gomso Bay, The Yellow Sea	Antibacterial (MRSA, MDRSA) DPPH radical-scavenging	[45]
16α-Methylaspochalasin J (**64**)	*Westerdykella dispersa*	The South China Sea	Antibacterial *(Bacillus subtilis)*	[46]
16-Hydroxymethylaspergillin PZ (**65**)			Antibacterial *(Proteus vulgaris*, *Enterobacter aerogenes)*	
2-Hydroxy-6-((1E,3E)-7-hydroxyundeca-1,3-dienyl)benzaldehyde (**66**)	*Zopfiella marina* BCC 18240 (or NBRC 30420)	coast of Taiwan	Antibacterial *(Mycobacterium tuberculosis* H37Ra, *Bacillus cereus)*	[47]
Asperfurandione A (**67**)	*Aspergillus versicolor*	Dongji Island, The East China Sea	Antifungal *(Gaeumannomyces graminis*, *Cryptococcus neoformans*, *Candida albicans)*	[48]
Asperfurandione B (**68**)				
Pleosporalone A (**69**),	*Pleosporales* sp. CF09-1	The Bohai Sea	Antigungal *(Botrytis cinerea*, *Rhizopus oryzae*, *Phytophthora capsica)*	[49,50]
Pleosporalone B (**70**)			Antifungal *(Alternaria brassicicola*, *Fusarium oxysporum)*	
Pleosporalone C (**71**)			Antifungal *(Botryosphaeria dothidea)*	
Ustusol A (**72**)			Antifungal *(Thielaviopsis paradoxa*, *Pestalotia calabae*, and *Gloeosporium musarum)*	[51]
12*R*,13*R*)-Dihydroxylanomycinol (**73**),	*Westerdykella dispersa*	The South China Sea	Antifungal *(Rhizoctorzia solani*, *Verticillium dahliae*, *Helminthosporium maydis*, *Fusarium tricinctum, F. oxysporum*, *Botryosphaeria dothidea*, and *Alternaria fragriae)*	[52]
(12*S*,13*S*)-Dihydroxylanomycinol (**74**)				
(12*R*,13*S*)-Dihydroxylanomycinol (**75**)				
(12*S*,13*R*)-Dihydroxylanomycinol (**76**)				
(12*S*,13*R*)-N-Acetyl-dihydroxylanomycin (**77**)				
(12S,13S)-N-Acetyl-dihydroxylanomycin (**78**)				
Lanomycinol (**79**)				
Trichosordarin A (**80**)	*Trichoderma harzianum* R5	The Bohai Sea	Toxicity for plankton *(Artemia salina*, *Amphidinium carterae* and *Phaeocystis globose)*	
Chetracin E (**81**)	*Acrostalagmus luteoalbus* HDN13-530	Liaodong Bay, The Bohai Sea	Cytotoxicity, Inhibition of Hsp90	[57]
Chetracin F (**82**)				
Waikikiamide A (**83**)	*Aspergillus* sp. FM242	Waikiki beach, Oahu, Honolulu, Hawaii	Antiproliferative activity (HT1080, PC3, Jurkat, A2780 line cells)	[58]
Waikikiamide C (**84**)				
Asperienes A–D (**86**–**89**)	*Aspergillus flavus* CF13-11	The Bohai Sea	Cytotoxicity (HeLa, MCF-7, MGC-803 and A549 cell lines)	[59]
6*β*,9*α*,14-Trihydroxycinnamolide (**90**)	*Aspergillus flocculosus*	Nha Trang Bay, The South China Sea	Cytotoxicity (22Rv1, MCF-7, Neuro-2a)	[60]
Insulicolide A (**91**)				
Pyripyropene E (**92**)	*Aspergillus fumigatus* YK-7	Yingkou, The Bohai Sea	Cytotoxicity (U937 cell line)	[61]
Aurasperone H (**93**)	*Aspergillus niger* 2HL-M-8	Northeast Brazilian coast, The Atlantic Ocean	Cytotoxicity (A549, HL-60)	[62]
Methyl 5-(1-acetamido-2-phenylethyl)furan-2-carboxylate (**94**)	*Aspergillus niger*	Northeast Brazilian coast, The Atlantic Ocean	Cytotoxicity (HCT-116)	[63]
17-O-ethylnotoamide M (**95**)	*Aspergillus sulphureus* KMM 4640 and *Isaria felina* KMM 4639	East Sakhalin shelf, The Sea of OkhotskThe South China Sea, coast of Vietnam	Inhibition of the colony formation (22Rv1 cells)	[64]
Asperolide E (**96**)	*Aspergillus wentii* SD-310	The South China Sea	Cytotoxicity (HeLa, MCF-7, NCI-H446 line cells)	[65]
Variecolortin A (**97**)	*Eurotium* sp. SCSIO F452	The South China Sea	Cytotoxicity (SF-268, HepG2 cell lines)	[66]
Variecolortin B (**98**)				
Scopararane I (**99**)	*Eutypella* sp. FS46	The South China Sea	Cytotoxicity (MCF-7, NCI-H460, SF-268) - -	[67]
Hypoxone A (**100**)	*Hypoxylon rubiginosum* FS521	The South China Sea		
4,8-Dimethoxy-1-naphthol (**101**)				
1’-Hydroxy-4’,8,8’-trimethoxy[2,2’]binaphthalenyl-1,4-dione (**102**)			Cytotoxicity (SF-268, MCF-7, HepG-2, and A549)	[68]
Isariketide A (**103**)	*Isaria felina*	The South China Sea, coast of Vietnam	Cytotoxicity (human prostate cancer cells) -	[69]
Isariketide B (**104**)				
Mycophenolic acid (**105**)	*Penicillium brevicompactum* OUCMDZ-4920	The South China Sea	Cytotoxicity (P388, KB, HT29, MCF-7, A549)	[70]
Dicitrinone D (**106**)	*Penicillium citrinum*	Southeast coast of China	Cytotoxicity (SPC-A1)	[71]
Fusaperazine F (**107**)	*Penicillium crustosum* HDN153086	Prydz Bay, The Antarctic Ocean	Cytotoxicity (K562)	[72]
Penicisteroid E (**108**)	*Penicillium granulatum* MCCC 3A00475	Antarctica	Antiproliferative effect (12 cancer cell lines)s	[73]
Penicisteroid G (**109**)				
Penicisteroid H (**110**)				
Penicisteroid A (**111**)				
Penicisteroid C (**112**)				
Resorcinoside A (**113**)	*Penicillium janthinellum*	Cu Lao Cham Island, The South China Sea	Cytotoxicity (NUGC-3)	[74]
Secalonic acid H (**114**)	*Penicillum oxalicum* fungus (Langqi Island, Taiwan Strait)		Cytotoxicity (HCT116, KB, EC9706)	[75]
Secalonic acid I (**115**)				[75]
Secalonic acid J (**116**)			Cytotoxicity (HeLa, HCT116, MCF-7, Hep-3B, A549)	[76]
Secalonic acid K (**117**)				
Secalonic acid L (**118**)				
Secalonic acid M (**119**)				
Pestalone C (**120**) Pestalone E (**121**)	*Pestalotiopsis neglecta*	Gagedo Island, The Yellow Sea	Antiproliferative and apoptotic activity (PANC-1)	[77]
Tenellone H (**122**)	*Phomopsis lithocarpus* FS508	The Indian Ocean	Cytotoxicity (HepG-2, A549)	[78]
Lithocarols A–D (**123**–**126**)			Cytotoxicity (HepG-2, MCF-7, SF-268, and A549)	[79]
Lithocarpinols A (**127**) and B (**128**)				[80]
Dipleosporalones A (**129**) and B (**130**)	*Pleosporales* sp.	Coast of Huanghua, The Bohai Sea	Cytotoxicity (MDA-MB-231, MCF-7, MGC-803, HeLa, A549)	[81]
Auxarthrols D (**131**) and F (**132**)	*Sporendonema casei*	Zhangzi Island, The Yellow Sea	Cytotoxicity (a few human cancer cells)	[82]
Altersolanol B (**133**)			Antibacterial *(Mycobacterium phlei*, *Bacillus subtilis*, *Vibrio parahemolyticus* and *Pseudomonas aeruginosa)*	
9,10-Diolhinokiic acid (**134**)	*Talaromyces purpurogenus*	Coast of Qinghuangdao, The Bohai Sea	Cytotoxicity (HL-60 and A549)	[83]
Roussoellol C (**135**)			Cytotoxicity (MCF-7 and HL-60)	
Dankasterone (**136**)			Cytotoxicity (MCF-7 and HL-60)	
19-Methoxy-19,20-dihydrophomacin C (**137**)	*Westerdykella dispersa*	Guangzhou, China, The South China Sea	Cytotoxicity (MCF-7, HepG2, A549, HT-29 and SGC-7901)	[84]
Gymnastatin Z (**138**)				
Phomacin B (**139**)				
Cytochalasin P1 (**140**)	*Xylaria* sp. SOF11	The South China Sea	Cytotoxicity (MCF-7, SF-268)	[85]
19, 20-Epoxycytochalasin D (**141**)				
Acremeremophilanes B–F (**142**–**146**) and N (**147**)	*Acremonium* sp.	South part of The Atlantic Ocean	Inhibition of NO production	[88]
14-Hydroxy-cyclopeptine (**148**)	*Aspergillus* sp. SCSIOW2	The South China Sea	Inhibition of NO production	[89]
Dihydrobipolaroxin (**149**)				[90]
Dihydrobipolaroxin B (**150**)				
Dihydrobipolaroxin C (**151**)				
Dihydrobipolaroxin D (**152**)				
Asperteretal F (**153**)	*Aspergillus terreus* Y10	Coastal area of Hainan, The South China Sea	Inhibition of TNF-α generation	[91]
Asperversiamide G (**154**)	*Aspergillus versicolor*	The South China Sea	Inhibition of iNO synthase activity	[92]
6-[1-(2-aminobenzoyloxy)ethyl]-1-phenazinecarboxylic acid (**155**)	*Cystobasidium larynigs*	The Indian Ocean	Inhibition of NO production	[93]
Saphenol (**156**)				
Saphenic acid (**157**)				
Phenazine-1-carboxylic acid (**158**)				
6-(1-Hydroxyehtyl)phenazine-1-carboxylic acid (**159**)				
6-Acetylphenazine-1-carboxylic acid (**160**)				
Khusinol B (**161**)	*Graphostroma* sp. MCCC 3A00421	The Atlantic Ocean	Inhibition of NO production	[94]
Emodacidamides A (**162**), C–E (**163**–**165**)	*Penicillium* sp. SCSIO sof101		Inhibition of interleukin-2 secretion	[95]
7-Acetoxydehydroaustinol (**166**)	*Penicillium* sp. F-5497	Busan, South Korea, Korean Strait	Suppression of NO overproduction	[96]
Chrysamide C (**167**)	*Penicillium chrysogenum* SCSIO41001	The Indian Ocean	Suppression of interleukin-17 production	[97]
Neuchromenin (**168**)	*Penicillium glabrum* SF-7123	The Ross Sea, Antarctica	Inhibition of NO and prostaglandin E2 production	[98]
Myxotrichin C (**169**)				
Deoxyfunicone (**170**)				
Restricticin B (**171**)	*Penicillium janthinellum*	Cu Lao Cham Island, The South China Sea	Suppression of pro-inflammatory mediators production	[99]
N-acetyl restricticin (**172**)				
Piltunine E (**173**)	*Penicillium piltunense* KMM 4668	Sakhalin Island, The Sea of Okhotsk	Downregulation of ROS production	[100]
5′-Hydroxyasperentin (**174**)				
			Decreasing of NO production	
Dehydrocurvularin (**175**)	*Penicillium sumatrense*	The Indian Ocean	Inhibition of NO production	[101]
Citrinin H1 (**176**)	*Penicillium* sp. SF-5629	Ulgin, South Korea, The Sea of Japan	Inhibition of NO and prostaglandin E2 production	[102]
Arthone C (**177**)	*Arthrinium* sp. UJNMF0008	The South China Sea	DPPH- and ABTS-radical scavenging	[104]
6,9-Dibromoflavasperone (**178**)	*Aspergillus niger*	Suncheon Bay, South Korea, Korean Strait	DPPH-radical scavenging	[105]
Flavasperone (**179**)				
TMC-256A1 (**180**)				
Fonsecin (**181**)				
Aurasperone B (**182**)				
Oxisterigmatocystin D (**183**)	*Aspergillus versicolor*	The South China Sea	Antioxidant activities in TEAC assay	[106]
Oxisterigmatocystin C (**184**)			Antioxidant activities in TEAC assay	
Sterigmatocystine (**185**)				
Dihydrosterigmatocystine (**186**)				
Versicolorin B (**187**)				
UCT1072M1 (**188**)			Antioxidant activities in TEAC assay, Activation of Nrf2-regulated gene expression	
Averantin (**189**)				
Methylaverantin (**190**)			Antioxidant activities in TEAC assay	
Averythrin (**191**)			Antioxidant activities in TEAC assay, Activation of Nrf2-regulated gene expression	
Averufanin (**192**)			Antioxidant activities in TEAC assay	
Averufine (**193**)				
Nidurufin (**194**)			Antioxidant activities in TEAC assay, Activation of Nrf2-regulated gene expression	
Cladosporin D (**195**)	*Cladosporium* sp. SCSIO z015	Okinawa Trough, The East China Sea	DPPH radical scavenging	[107]
Eurotiumins A–C (**196**–**198**)	*Eurotium* sp. SCSIO F452	The South China Sea	DPPH-radical scavenging	[108]
Eurotinoids A–C (**199**–**204**)				[109]
(+)-Dihydrocryptoechinulin D (**205**)			Cytotoxicity (SF-268, HepG2) DPPH-radical scavenging	
(-)-Dihydrocryptoechinulin D (**206**)				
(+)-Euroticin B (**207**)			Antioxidant activities	[110]
(–)-Euroticin B (**208**)				
Niveoglaucin A (**209**)	*Aspergillus niveoglaucus*	Nha Trang Bay, The South China Sea	Suppression of ROS hyperproduction, Neuroprotective activity	[111]
Flavoglaucin (**210**)				[111]
(+)-Cryptoechinuline B (**211**)				[112]
(–)-Cryptoechinuline B (**212**)				
Neoechinulin (**213**)				
Neoechinulines B–C (**214**–**215**)				
Perylenequinone (**216**)	*Alternaria* sp. NH-F6	The South China Sea	Inhibition of BRD4 protein	[122]
Protuboxepin K (**217**)	*Aspergillus* sp. BFM-0085	Tokyo Bay, Tokyo, Japan	Inhibition of (BMP)-induced alkaline phosphatase activity	[123]
Protuboxepin A (**218**)				
Flavipesolides A–C (**219**–**221**)	*Aspergillus flavipes* HN4-13	The Yellow Sea	Inhibition of α-glucosidase activity	[124]
5-[(3,4-Dihydro-2,2-dimethyl-2H-1-benzopyran-6-yl)methyl]-3- hydroxy-4-(4-hydroxyphenyl)-2(5H)furanone (**222**)				
Aspernolide A (**223**)				
Emodin (**224**)				
Geodin hydrate (**225**)				
Methyl dichloroasterrate (**226**)				
Monomethylosoic acid (**227**)				
Asperversin G (**228**)	*Aspergillus versicolor*	The South China Sea	Inhibition of AChE activity	[125]
7-Chlorofolipastatin (**229**)	*Aspergillus ungui*	Suruga Bay, Japan	Inhibition of SOAT1- and SOAT2-expressing	[126]
Biscogniauxone (**230**)	*Biscogniauxia mediterranea*	Herodotes Deep, The Mediterranean Sea	Inhibition of glycogen synthase kinase (GSK-3β) activity	[127]
Cyclo-(L-Phe-L-Leu-L-Val-L-Leu-L-Leu) (**231**)				
Preaustinoid A6 (**232**)	*Penicillium* sp. SF-5497	Gijang-gun, Busan, South Korea, Korean Strait	Inhibition of PTP1B activity	[128]
Berkeleyone C (**233**)				
Butanolide A (**234**)	*Penicillium* sp. S-1-18	Antarctica		[129]
Austalide V (**235**)	*Penicillium rudallense*	Ga-geo Island, South Korea, The Yellow Sea	Inhibition of (RANKL)-induced osteoclast differentiation	[130]
Austalide W (**236**)				
Diorcinol J (**237**)	*Aspergillus sulphureus* KMM 4640 (The Sea of Okhotsk) and *Isaria felina* KMM 4639 (The South China Sea)		Hemolytic activity and cytotoxicity (Ehrlich carcinoma)	[131]
Diorcinol B (**238**)			Enhancing of Hsp70 expression	
Terretonin H (**239**)	*Aspergillus ustus* KMM 4664	The Sea of Okhotsk	Inhibition of the ability of spermatozoa to fertilize egg-cells of the sea urchin *Strongilocentrotus intermedius*	[132]
Terretonin I (**240**)				
*ent*-Peniciherqueinone (**241**)	*Penicillium* sp.	Gagudo, South Korea	Induction of adipogenesis	[133]
4-Hydroxysclerodin (**242**)			Anti-angiogenetic and anti-inflammatory activities, Inhibition of tube formation	
(9R)-2,5,7-Trihydroxy-1,8,8,9-tetramethyl-5-(2-oxopropyl)-8,9-dihydro-3H-phenaleno[1,2-b]furan-3,4,6(3aH,5H)-trione (**243**)			Inhibition of NO production	
Isoherqueinone (**244**)			Induction of adipogenesis	
Spirograterpene A (**245**)	*Penicillium granulatum* MCCC 3A00475		Antiallergic activity	[134]
Stachybotrysin (**246**)	*Stachybotrys* sp. KCB13F013	Wi Island, South Korea, The Yellow Sea	Inhibition of osteoclast differentiation	[135]

Acronyms: SA—*Staphylococcus aureus*, MRSA—methicillin-resistant *Staphylococcus aureus*, E. coli—*Escherichia coli*, VRE—vancomycin-resistant *Enterococcus faecalis*, MDRSA—multidrug-resistant *Staphylococcus aureus*.
